# From molecular mechanisms to clinical practice: advances in the immune microenvironment and translational therapeutics of colorectal cancer liver metastasis

**DOI:** 10.3389/fimmu.2026.1934944

**Published:** 2026-10-30

**Authors:** Ruiqi Zhang, Jie Qu, Hongfang Chi, Zhiqiang Li, Farui Zhang

**Affiliations:** Tianshui Traditional Chinese Medicine Hospital, Department of General Surgery, Tianshui, China

**Keywords:** colorectal cancer liver metastasis, gut microbiota, immune checkpoint inhibitors, metabolic reprogramming, pre-metastatic niche, tumor microenvironment

## Abstract

Colorectal cancer (CRC) ranks third in global incidence and second in cancer-related mortality, with the liver constituting the most frequent target organ for metastatic dissemination and distant organ involvement representing the predominant cause of death. Approximately 20–25% of patients present with synchronous hepatic metastases at initial diagnosis or within 3–6 months thereafter, whereas an additional 20–50% develop metachronous hepatic recurrence following curative-intent resection of the primary tumor, thereby positioning colorectal cancer liver metastasis (CRLM) among the most formidable challenges in oncology. With progressive advances in biological insight, the conceptual framework of CRLM has evolved from the classical ‘seed and soil’ hypothesis into an integrative, multi-dimensional paradigm encompassing the epithelial-mesenchymal transition (EMT) of circulating tumor cells (CTCs), immunostromal crosstalk within the tumor microenvironment (TME), the remote regulatory functions of the gut microbiota and its metabolic products, and the contributions of metabolic reprogramming and epigenetic modifications. This review synthesizes recent advances in the molecular pathogenesis of CRLM, including the formation and modulation of the pre-metastatic niche (PMN), the influence of liver-intrinsic immune tolerance on immunotherapeutic responsiveness, the synergistic regulation of core signaling networks, the pathogenic role of the gut microbiota–metabolism–epigenetics axis, and the role of CTCs within the metastatic cascade. From a clinical perspective, we appraise the progress in surgical resection, immune checkpoint inhibitors (ICIs) and their combination strategies, targeted therapies, locoregional interventions, and emerging metabolic–epigenetic therapeutics, with particular emphasis on the clinically challenging disparity in immunotherapy responses between hepatic and pulmonary metastases, a phenomenon largely attributable to organ-specific immune microenvironments. Furthermore, we delineate the limitations of current investigations, noting that much of the mechanistic evidence derives from preclinical models and that organ-specific clinical observations remain predominantly retrospective in nature, and We emphasize that developing precision treatment strategies based on multidimensional molecular profiling and organ-specific therapeutic regimens will constitute a critical research direction for improving the prognosis of patients with CRLM.

## 1 Introduction

Colorectal cancer (CRC) constitutes a substantial threat to global public health. According to the 2022 global cancer statistics (GLOBOCAN), CRC accounts for approximately 1.9 million new incident cases (9.6% of all malignancies) and 0.9 million annual deaths (9.3% of total cancer mortality), ranking third and second in global incidence and mortality, respectively (1). Compounding this burden, the incidence of CRC continues to escalate in low- and middle-income countries alongside economic development and the westernization of lifestyle, while a concerning upward trend is simultaneously observed in younger populations (2). These epidemiological features prioritize the prevention and treatment of CRC as a global oncological imperative.

Distant metastasis represents the principal cause of CRC-related death. Owing to its unique anatomical position and specialized immunological milieu, the liver constitutes the most frequent target organ for metastatic dissemination. Epidemiological data indicate that approximately 20–25% of patients harbor synchronous hepatic metastases at initial presentation, whereas 20–50% of those with initially localized disease ultimately develop metachronous liver metastases (3). Synchronous CRLM is defined as hepatic metastasis detected at initial diagnosis or within 3–6 months thereafter, whereas metachronous CRLM refers to metastasis detected more than 6 months following curative-intent resection. Hepatic involvement accounts for approximately 49% of all CRC-attributable mortality (4). Currently, radical surgical resection remains the only therapeutic modality offering the prospect of long-term survival for patients with CRLM, with post-operative 5-year survival rates reaching 30–57% (5). However, only approximately 10–20% of patients present with resectable disease at diagnosis (6); the vast majority are ineligible for curative surgery due to excessive hepatic tumor burden, insufficient future liver remnant, or significant comorbid conditions, resulting in a 5-year survival rate of less than 5% (7). This stark clinical reality underscores the urgency and critical importance of elucidating the mechanisms driving CRLM, optimizing early diagnostic strategies, and developing novel therapeutic modalities.

Since Stephen Paget articulated the archetypal ‘seed and soil’ hypothesis in 1889 (8), the theoretical framework governing metastasis research has undergone continuous evolution. This hypothesis posits that successful distant colonization by tumor cells depends not only on the intrinsic invasive capacity of the ‘seed’ but, critically, on the permissive state of the target organ microenvironment, or the ‘soil’. This foundational insight has been substantiated and substantially extended by contemporary CRLM research. Modern tumor biology has unveiled a more intricate regulatory landscape: prior to the detachment of tumor cells, primary CRC actively ‘educates’ the hepatic microenvironment through the secretion of soluble factors and extracellular vesicles (EVs), thereby forming the so-called pre-metastatic niche (PMN) (9). Upon arrival in the liver, circulating tumor cells (CTCs) engage in complex interactions with resident hepatic cells-including liver sinusoidal endothelial cells, Kupffer cells, and hepatic stellate cells-as well as recruited immunostromal populations, collectively constructing the metastatic tumor microenvironment (TME). This process is coordinately regulated across multiple dimensions: tumor metabolic reprogramming, exemplified by Warburg effect-driven lactate accumulation, reshapes the gene expression landscape through epigenetic modifications, particularly lactylation (10); the gut microbiota and its metabolic outputs remotely participate in the immunomodulation of the TME via the gut–liver axis (11); and the inherent immunotolerant properties of the liver confer a unique capacity for immune escape upon hepatic metastases (12).

Of particular significance, recent clinical investigations have revealed a phenomenon of translational importance: the anatomical site of metastasis significantly influences the efficacy of immunotherapy. Subgroup analyses from multiple clinical trials and retrospective studies consistently demonstrate that patients with liver metastases exhibit a markedly inferior response rate to immune checkpoint inhibitors (ICIs) compared with those with pulmonary metastases, a discrepancy that persists even within the microsatellite instability-high (MSI-H)/mismatch repair-deficient (dMMR) subgroup that typically derives the greatest benefit (13, 14). This finding not only provides critical guidance for clinical decision-making but also suggests that the organ-specific immune microenvironment plays a decisive role in determining therapeutic response, thereby providing a rationale for the development of organ-tailored therapeutic strategies.

Against this backdrop, this review aims to systematically synthesize advances in the mechanistic understanding and clinical management of CRLM. At the mechanistic level, we organize the discourse around an integrative ‘seeds–soil–environmental modifiers’ framework, dissecting the EMT process of CTCs, the cellular and molecular networks of the TME, and the regulatory mechanisms of the gut microbiota and metabolic reprogramming (**Table 1**). At the clinical level, we comprehensively evaluate the current status and challenges of strategies encompassing surgery, immunotherapy, targeted therapy, and locoregional interventions. Finally, we delineate the principal limitations of current research and offer a perspective on critical future research directions.

**Table 1:** Evidence map of the ”seed-soil-environmental modifiers” framework.

Framework element	Representative interaction	Evidence source	Nature of evidence	Key limitation	Reference
Seed	Association between CTC count/molecular features and CRLM prognosis	Human CRLM liquid-biopsy cohorts	Associative (human)	Assay not standardized; causality undetermined	(15)
Seed	CTC-EMT promotes hepatic metastatic colonization	*In vitro* + mouse	Causal (model); hypothetical (human)	Hybrid EMT/plasticity states exist; EMT not universally required	(16, 17)
Seed	Platelet cloaking and NET-mediated capture of CTCs	*In vitro* + mouse	Causal (model); indirect (human)	No functional validation in humans	(18–20)
Soil	Primary-tumor EVs/factors remodel the hepatic PMN	Mainly mouse	Causal (mouse); hypothetical (human)	Human PMN cannot be serially monitored	(9, 21–23)
Soil	Reprogramming of HSCs/LSECs/Kupffer cells	Human tissue + mouse	Causal (mouse); associative (human)	Human tissue largely reflects static assessment of established metastases	(24–27)
Soil	Hepatic immune tolerance → primary ICI resistance	Human retrospective subgroups + mouse mechanisms	Associative (human); causal (mouse)	Multiple confounders; lack of prospective validation	(13, 14, 28, 29)
Environmental modifier	Gut microbiota and metabolites promote liver metastasis	Human cohorts/tissue + mouse	Associative (human); causal (mouse)	Confounded by sample source, antibiotics, diet, geography	(30–32)
Environmental modifier	Lactylation as a metabolic–epigenetic bridge	Human CRC tissue/cell lines; limited direct CRLM evidence	Hypothetical (insufficient human CRLM evidence)	Insufficient CRLM-specific human evidence	(10, 33, 34)
Environmental modifier	Glucose/lipid metabolic reprogramming supports metastatic growth	Human tissue + mouse	Causal (mouse); associative (human)	Organ-specific metabolic differences inadequately characterized	(35–38)

The literature search strategy underlying this review was designed to ensure broad thematic coverage and representativeness. The databases searched included PubMed, Web of Science, and Embase, with a cutoff date of February 2026. The core English-language search combined Medical Subject Headings (MeSH) with free-text terms, using the primary search string: (“colorectal cancer” OR “colorectal carcinoma” OR “colon cancer” OR “rectal cancer”) AND (“liver metastasis” OR “hepatic metastasis”), which was subsequently expanded with topic-specific terms such as “pre-metastatic niche,” “tumor microenvironment,” “immune checkpoint inhibitors,” “circulating tumor cells,” “epithelial-mesenchymal transition,” “metabolic reprogramming,” “lactylation,” “gut microbiota,” and “organ-specific immunity.” Eligible literature comprised original investigations, reviews, systematic reviews/meta-analyses, and key clinical practice guidelines. In terms of evidence prioritization, studies published within the preceding five years in high-impact peer-reviewed journals were preferentially cited, while milestone discoveries of seminal importance were also incorporated; for mechanistic sections, studies with complete experimental evidence chains and clear mechanistic delineation were favored, whereas for clinical sections, large-scale randomized controlled trials, authoritative guidelines, and high-quality meta-analyses were prioritized. Given that this review is narrative in nature and aims to systematically integrate mechanistic and clinical advances in CRLM rather than to perform quantitative synthesis, the rigorous exclusion criteria of a systematic review were not adopted; nonetheless, the described search strategy ensures transparency and reproducibility of the literature source. Inclusion was restricted to original studies, reviews, systematic reviews/meta-analyses, and clinical practice guidelines published in peer-reviewed journals; conference abstracts, case reports, editorials, and non-peer-reviewed sources were excluded.

## 2 Molecular mechanisms of colorectal cancer liver metastasis

### 2.1 Anatomical foundations: physical predisposition of the liver as a metastatic target

The propensity of CRC to metastasize to the liver is primarily predicated on the unique anatomical configuration and hemodynamic characteristics that constitute the physical basis for CRLM (**Figure 1**). The venous drainage of the colon sequentially converges into the superior and inferior mesenteric veins, culminating in direct perfusion of the liver via the main portal vein. This ‘mesenteric-portal’ circulatory pathway provides a naturally expedited transit route for the dissemination of intestinal tumor cells to the liver. Invasive CRC cells, having breached the mucosal, muscular, and vascular basement membrane barriers of the bowel wall, can directly invade the mesenteric venous tributaries, thereby continuously inundating the hepatic microcirculation with portal venous flow, which substantially increases the probability of intrahepatic arrest and colonization.

**Figure 1:**
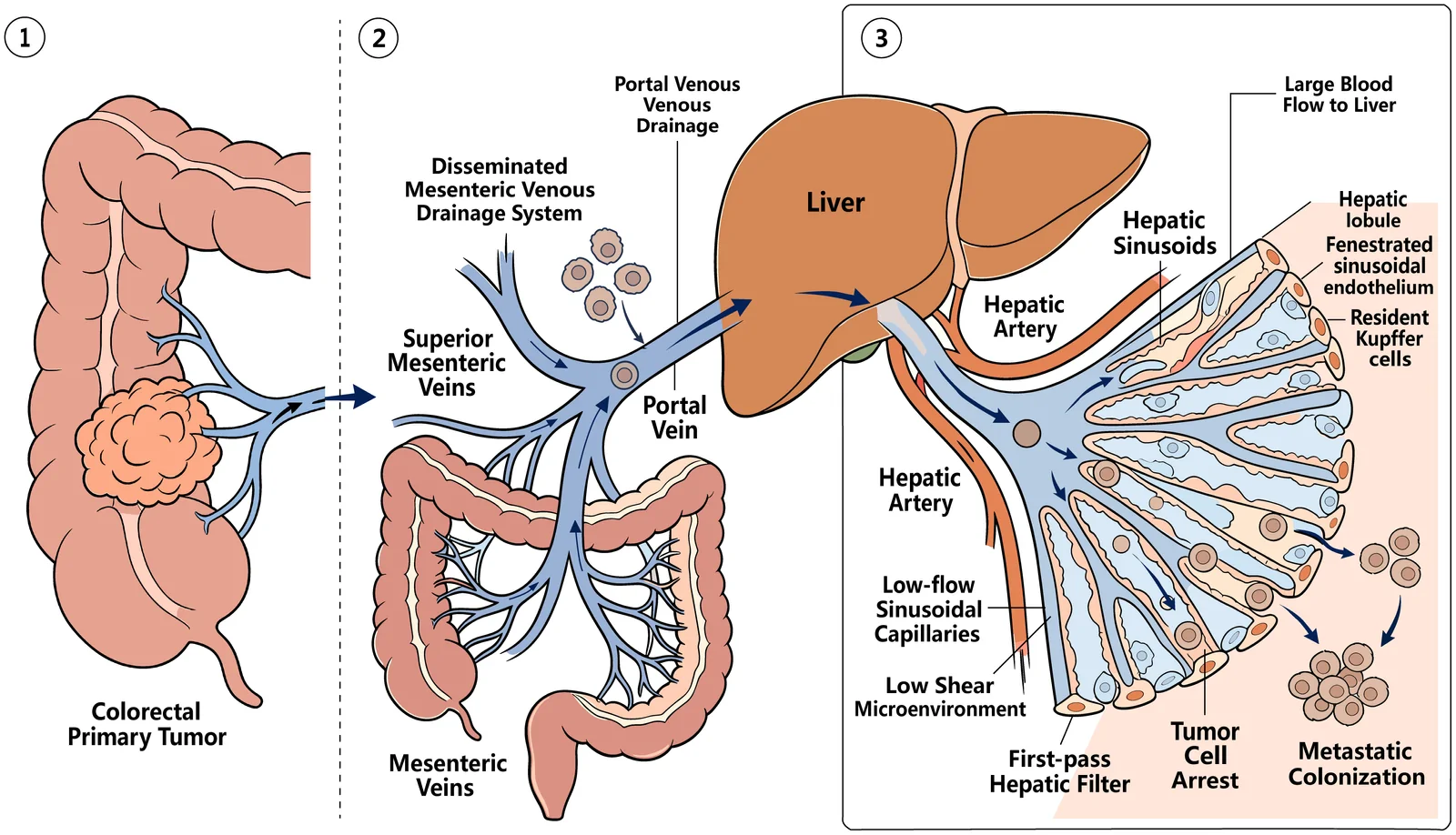
Anatomical foundations underlying the propensity for colorectal cancer liver metastasis. The invasion of primary colorectal carcinoma into the mesenteric-portal venous network establishes a direct hematogenous conduit through which tumor cells are transported into the hepatic circulation. Within the sinusoidal microvasculature, a microenvironment characterized by low-velocity blood flow and a discontinuous, fenestrated endothelium facilitates the first-pass mechanical arrest of circulating tumor cells, thereby creating a permissive physical substrate for their subsequent extravasation, survival, and colonization within the hepatic parenchyma. Evidence level: The anatomical pathways and hemodynamic characteristics are established facts in human anatomy and physiology; among them, the proposition that "first-pass trapping facilitates metastatic colonization" constitutes a mechanistic inference derived from anatomical observations and animal model studies. Causal direction: The anatomical pathway acts as a physical prerequisite for the occurrence of hepatic metastasis, but it does not represent a sufficient condition for this pathological process.

From the perspective of hepatic microarchitecture, the sinusoid constitutes the critical locus for material exchange and transvascular cell migration. Liver sinusoidal endothelial cells (LSECs) are characterized by a thin, discontinuous cytoplasm, spacious fenestrations, and a lack of a complete basement membrane, conferring a permeability that is significantly higher than that of other systemic vascular beds. This structural characteristic facilitates the relatively easy traversal of the vascular wall by CTCs, enabling their entry into the Space of Disse and subsequent extravasation and early colonization within the hepatic parenchyma. Simultaneously, the liver, being the body’s largest metabolic and detoxification organ, possesses an exceptionally rich blood supply and relatively slow blood flow, which furnishes arrested tumor cells with abundant oxygen, nutrients, and growth factors, satisfying the energetic and biosynthetic demands for initial proliferation and subsequent micrometastatic expansion. Furthermore, the dual blood supply to the liver-receiving approximately 75% of inflow from the portal vein and 25% from the hepatic artery-ensures adequate perfusion for the sustained growth and angiogenesis of metastatic foci, enabling clinically occult micrometastases to progressively evolve into radiologically discernible solid tumors.

In summary, the anatomical features rooted in the venous drainage pattern of the colon, the specialized ultrastructure of the hepatic sinusoids, the abundant blood flow, and the dual vascular supply collectively establish the physical foundation for the high incidence and organ selectivity of CRLM. These anatomical conditions not only explain the predilection of CRC for hepatic metastasis but also provide the indispensable physical substrate upon which the complex molecular dialogues and cellular regulations between tumor cells and the intrahepatic microenvironment subsequently unfold.

### 2.2 Formation and regulation of the pre-metastatic niche

The concept of the pre-metastatic niche (PMN) was first systematically articulated by Kaplan et al. in 2005, with the seminal finding that VEGFR1+ bone marrow-derived hematopoietic progenitor cells cluster at future sites of metastasis prior to the arrival of tumor cells (21). This observation indicates that distant metastasis is not a consequence of stochastic tumor cell dissemination but rather a process whereby the primary tumor remodels the target organ microenvironment through secreted factors, creating conditions permissive for subsequent metastatic colonization. In CRC, PMN formation entails the coordinated involvement of tumor-derived soluble factors, extracellular vesicles (EVs), vascular remodeling, the recruitment of immunosuppressive cells, and the restructuring of the extracellular matrix (ECM) (9) (**Figure 2**).

**Figure 2:**
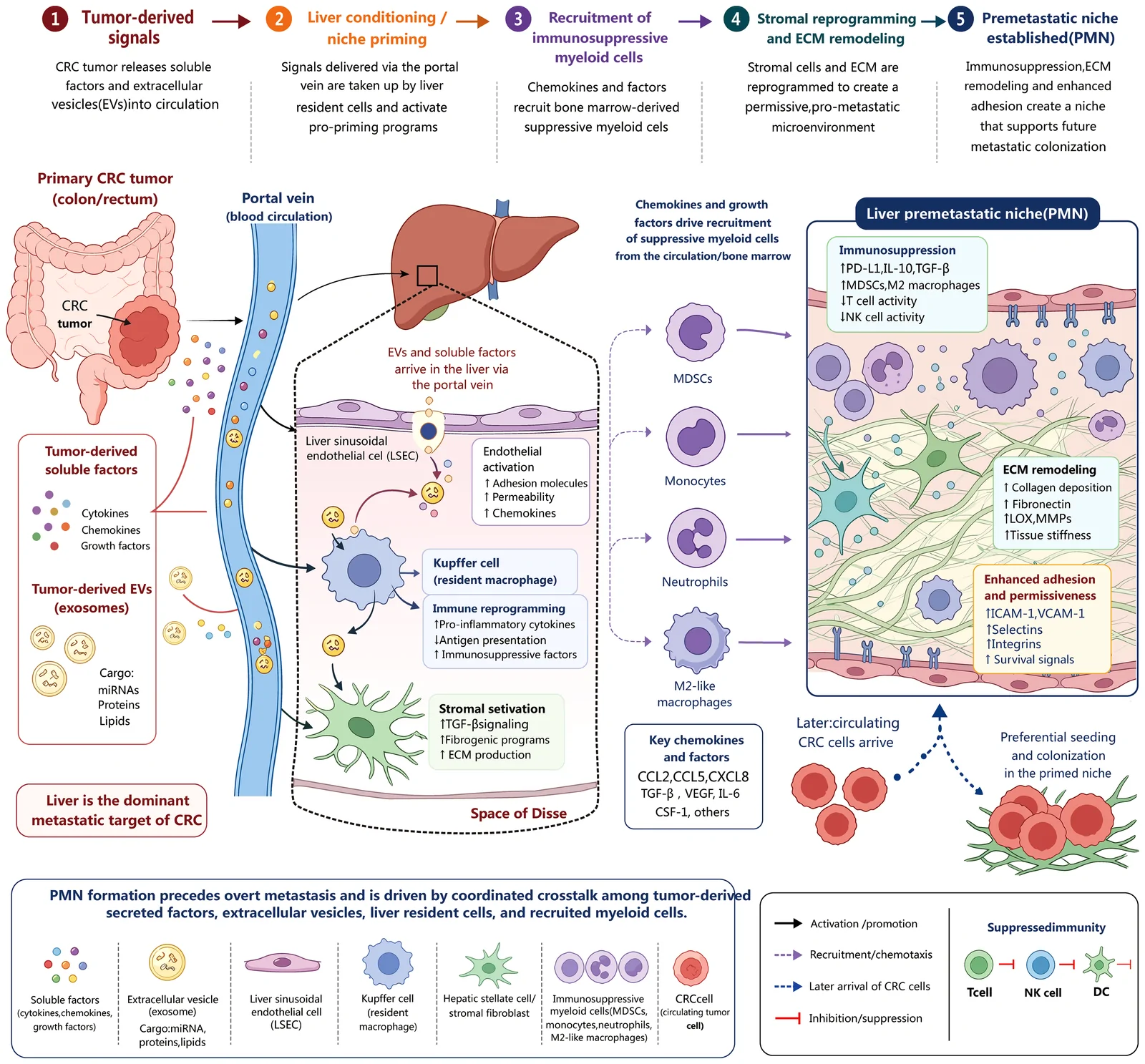
Mechanisms underpinning the formation of the hepatic pre-metastatic niche driven by colorectal cancer. Primary tumor-derived molecular signals orchestrate the activation of resident hepatic cells, coordinate the recruitment of immunosuppressive myeloid populations—including myeloid-derived suppressor cells and tumor-associated neutrophils—and instigate extensive remodeling of the hepatic stromal architecture, thereby pre-establishing an immunosuppressive, metastasis-permissive niche that facilitates the subsequent arrest, survival, and colonization of circulating tumor cells within the liver parenchyma.Evidence level: Evidence is primarily derived from animal models, while human-derived evidence comprises indirect cross-sectional evidence obtained from established metastatic lesions and peritumoral tissues. Causal direction: In mouse models, there exists temporal causal evidence supporting that primary tumor-derived signals induce hepatic microenvironment remodeling; however, in human subjects, the temporal sequence that "pre-metastatic niches form prior to the establishment of metastatic lesions" has not been directly verified.

Within the theoretical framework of the PMN, distinguishing between two pathogenic sources is of critical academic significance: the first comprises soluble factors and EVs derived from the primary tumor that exert biological functions prior to the onset of metastatic dissemination; the second comprises the systemic inflammatory response induced by established occult metastatic foci following dissemination. These two sources overlap temporally and are difficult to distinguish through static samples from clinical patients; consequently, existing PMN studies predominantly focus on the first category, namely the remote remodeling of the hepatic microenvironment by primary tumor-derived signals. Specifically, during the early stages of tumorigenesis, tumor cells and associated stromal cells within the primary lesion can continuously secrete soluble factors and release EVs carrying functional nucleic acids and proteins, which migrate to the liver via the systemic circulation and initiate the malignant remodeling of the intrahepatic microenvironment; distinct EV subsets can anchor to specific hepatic cell populations through different molecular recognition mechanisms, exerting differential regulatory functions, thereby pre-establishing a suitable ‘soil’ for the subsequent hepatic colonization of circulating tumor cells (CTCs). This constitutes the central focus of current research on PMN formation and regulation.

#### 2.2.1 Tumor-derived secreted factors and extracellular vesicles

The formation of the PMN is initiated by the release of a diverse array of soluble factors from the primary colorectal carcinoma. These factors include vascular endothelial growth factor-A (VEGF-A), transforming growth factor-β (TGF-β), tumor necrosis factor-α (TNF-α), multiple interleukins (ILs), and members of the S100 protein family (39). Among them, VEGF-A not only induces tumor angiogenesis but also stimulates tumor-associated macrophages (TAMs) to produce C-X-C motif chemokine ligand 1 (CXCL1), which accumulates within the hepatic parenchyma and recruits CXCR2+ myeloid-derived suppressor cells (MDSCs) to the developing PMN (40). Collectively, these factors exert profound effects on the vasculature of the target organ by upregulating the expression of endothelial adhesion molecules and promoting neovascularization, thereby establishing an early permissive microenvironment that facilitates subsequent tumor cell adhesion (9).

Extracellular vesicles (EVs), particularly exosomes, serve a messenger-like function in PMN formation. Tumor cell-derived EVs carry bioactive molecules, including proteins, messenger RNA (mRNA), microRNA (miRNA), and lipids, which are transported via the systemic circulation to the liver and, upon internalization by resident cells, alter their functional state. In a seminal study, Zeng et al. (22) demonstrated that CRC cell-derived exosomal miR-25-3p targets the transcription factors Krüppel-like factor 2 (KLF2) and Krüppel-like factor 4 (KLF4) in endothelial cells, resulting in the downregulation of the tight junction proteins zonula occludens-1 (ZO-1), occludin, and claudin-5. This effect compromises the integrity of the endothelial barrier and increases vascular permeability, thereby promoting both the extravasation of CTCs and PMN formation.

The organotropism of exosomes is largely determined by their surface integrin repertoire. In a landmark study, Hoshino et al. (41) established that exosomal integrins α6β4 and α6β1 are associated with pulmonary metastatic propensity, whereas integrin αvβ5 directs preferential exosomal homing to the liver. In the context of CRC, tumor-derived exosomes carrying αvβ5 are preferentially internalized by hepatic Kupffer cells, thereby initiating a liver-specific program of PMN formation. This finding provides a conceptual framework for understanding metastatic organotropism and suggests that blockade of specific integrins may represent a potential avenue for the prevention of organ-specific metastasis; however, the safety, efficacy, and feasibility of such interventional strategies in humans remain to be established.

CRC-derived exosomes further reshape the hepatic immune microenvironment through the delivery of specific miRNAs and proteins. Zhao et al. (42) found that CRC cell-derived exosomal miR-934 downregulates phosphatase and tensin homolog (PTEN) expression and activates the PI3K/AKT signaling pathway to induce an immunosuppressive pro-tumorigenic phenotype in macrophages, which subsequently secrete CXCL13 and activate a CXCL13/CXCR5/NF-κB/miR-934 positive feedback loop that further accelerates CRC progression and hepatic metastasis. Similarly, exosomal heat shock protein 90B1 (HSP90B1) promotes the conversion of hepatic macrophages from a pro-inflammatory phenotype toward an immunosuppressive pro-tumorigenic phenotype while concurrently reducing CD8+ T cell activity, thereby reinforcing the intrinsic immunotolerant state of the liver (43). In addition, the metalloproteinase ADAM17 carried by exosomes facilitates tumor cell migration through the cleavage of E-cadherin (44), whereas exosomal miR-21 activates Toll-like receptor 7 (TLR7) signaling in hepatic macrophages, triggering IL-6 production and driving the expansion of myeloid-derived suppressor cells (MDSCs), which further suppresses T cell responses (45).

#### 2.2.2 Recruitment of immunosuppressive myeloid cells

A hallmark event in PMN formation is the early accumulation of bone marrow-derived immunosuppressive cells within the target organ. Myeloid-derived suppressor cells (MDSCs) represent a heterogeneous population of immature myeloid cells endowed with potent immunosuppressive capacity. In CRC, tumor-derived tissue inhibitor of metalloproteinases-1 (TIMP-1) promotes the recruitment of neutrophils to the liver by upregulating the expression of stromal cell-derived factor-1 (SDF-1/CXCL12), thereby facilitating the establishment of the PMN (46). Lin et al. (47) further demonstrated that sustained activation of sphingosine-1-phosphate receptor 1 (S1PR1) induces the expansion and accumulation of MDSCs in the liver through STAT3 signaling; this process, in turn, upregulates IL-6 expression, which activates the JAK/STAT3 pathway in hepatocytes and establishes a positive feedback loop that further reinforces MDSC recruitment. Concurrently, tumor-derived VEGF-A stimulates TAMs to produce CXCL1, generating a chemotactic gradient that directs CXCR2+ MDSCs toward the hepatic PMN (40).

In recent years, the role of neutrophils within the PMN has attracted particular attention. Although neutrophils have traditionally been defined as effector cells mediating anti-infective immunity, within the tumor microenvironment these cells can polarize toward a pro-tumorigenic functional phenotype, commonly designated as the “N2-like” phenotype. It should be emphasized, however, that the N1/N2 dichotomy is likewise derived predominantly from *in vitro* polarization models and therefore constitutes a simplified, pedagogically oriented classification framework that cannot fully capture the continuous, heterogeneous, and microenvironment-dependent activation states of tumor-associated neutrophils (TANs) *in vivo*; the pro- or anti-tumorigenic activities of distinct TAN subsets may undergo dynamic conversion in response to microenvironmental signals, and consequently it is inappropriate to homogenize all neutrophils possessing pro-tumorigenic functions into a single “N2” population. TANs with pro-tumorigenic properties can release neutrophil extracellular traps (NETs) and secrete proteases to remodel the extracellular matrix, thereby providing microenvironmental support for tumor cell adhesion and survival (48). NETs are reticular structures assembled from decondensed chromatin and granular proteins, and in addition to their innate immune function of pathogen capture, they have been shown to capture CTCs, supplying a physical scaffold for the retention of CTCs within the hepatic sinusoids (49). NET formation is dependent on peptidylarginine deiminase 4 (PAD4)-mediated histone citrullination (50); accordingly, it has been demonstrated that inhibition of PAD4 activity or degradation of NETs by deoxyribonuclease I (DNase I) effectively suppresses the initiation and progression of CRC liver metastasis (51).

Within the hepatic PMN of CRLM, MDSCs and TANs act synergistically (52). The primary tumor recruits MDSCs to the liver through chemokine-mediated signaling, whereupon MDSCs secrete TGF-β and IL-10 to suppress CD8+ T cell function and induce ferroptosis-related immune escape, thereby establishing an immunosuppressive microenvironment. Simultaneously, TANs polarize toward a pro-tumorigenic phenotype under tumor-derived signals, providing a physical scaffold for CTCs through NET release and promoting tumor colonization via matrix remodeling and the amplification of local inflammation. The immune tolerance mediated by MDSCs and the structural support driven by TANs are mutually reinforcing, collectively constructing an intrahepatic microenvironment conducive to metastatic establishment.

#### 2.2.3 Reprogramming of hepatic stromal cells

The liver possesses a unique stromal cell composition, predominantly comprising hepatic stellate cells (HSCs), liver sinusoidal endothelial cells (LSECs), and Kupffer cells, all of which undergo profound functional reprogramming during PMN formation.

Hepatic stellate cells (HSCs) are liver-specific fibroblast-like cells residing in the Space of Disse, accounting for approximately 15% of non-parenchymal cells (53). In a physiological state, HSCs are quiescent and primarily function as vitamin A storage sites; however, under the influence of liver injury or tumor-derived signals, they are activated and transdifferentiate into a myofibroblast-like phenotype, producing copious amounts of extracellular matrix (ECM) components (particularly Type I and IV collagen) and various growth factors (54). CRC-derived exosomes play a critical role in this activation process. Yang et al. (23) demonstrated that CRC cell-derived extracellular vesicles are enriched with TGF-β1, which potently activates HSCs and promotes their differentiation into a cancer-associated fibroblast (CAF) phenotype. The activated HSC-derived CAFs highly express ECM proteins, such as fibronectin, which provide an ‘adhesive scaffold’ for the retention and colonization of tumor cells; they also secrete chemokines, such as CXCL12, to further recruit immunosuppressive myeloid cells, reinforcing the immune-exclusive features of the PMN (55).

Li et al. (24) further uncovered that CRC cell-derived exosomal miR-188-3p can directly activate HSCs, promoting the secretion of ECM components and establishing a metastasis-supportive intrahepatic microenvironment. Additionally, activated HSCs express PD-L1 and induce T cell dysfunction through the PD-1/PD-L1 pathway, further contributing to the immunotolerant hepatic environment (56). Another study by Zhao et al. (25) revealed a positive feedback loop wherein highly metastatic CRC cells release EVs rich in miR-181a-5p, which, by targeting and inhibiting SOCS3, activate the IL-6/STAT3 signaling pathway to sustain HSC activation. In turn, activated HSCs secrete CCL20, triggering a CCL20/CCR6/ERK1/2/ELK1/miR-181a-5p positive feedback circuit that continuously remodels the tumor microenvironment (TME) and promotes CRLM progression.

Liver sinusoidal endothelial cells (LSECs) are specialized endothelial cells lining the hepatic sinusoids, and their unique fenestrated architecture positions them as key regulators of material exchange and immunosurveillance. During PMN formation, LSECs undergo a series of functional alterations. Tumor-derived factors upregulate the expression of adhesion molecules such as E-selectin, vascular cell adhesion molecule-1 (VCAM-1), and intercellular adhesion molecule-1 (ICAM-1) on the LSEC surface, providing the molecular basis for the arrest and extravasation of CTCs in the liver (26). Moreover, under normal physiological conditions, LSECs contribute to maintaining hepatic immune tolerance via antigen presentation and the induction of regulatory T cells (Tregs) (57); within the PMN, this tolerogenic property is further amplified, with LSECs assisting tumor cells in evading T cell-mediated killing through programmed death-ligand 1 (PD-L1) expression and other inhibitory signals (26).

Kupffer cells are the resident macrophages of the liver, situated within the sinusoidal lumen and forming the first line of innate immune defense. During the early stages of metastasis, Kupffer cells are capable of eliminating the majority of tumor cells entering the liver through phagocytosis and the secretion of TNF-α and reactive oxygen species (58). However, tumor-derived signals, such as the interaction of exosomal integrin αvβ5 with Kupffer cells (41), can drive the transition of Kupffer cells from anti-tumor activity toward a pro-tumorigenic phenotype. The reprogrammed Kupffer cells/TAMs secrete VEGF, TGF-β, and IL-10, factors that not only promote tumor growth and angiogenesis but also reinforce the immunosuppressive environment by recruiting MDSCs and Tregs (27).

In summary, through the release of secreted factors, extracellular vesicles, and the mobilization of immune cells, CRC coordinately remodels the hepatic stroma, vasculature, and immune state, transforming a liver that is intrinsically unfavorable to tumor growth into a PMN characterized by immunosuppression, fibrosis, and heightened vascular permeability. This pre-established ‘soil’ creates a critical window for the subsequent survival and extravasation of CTCs. However, the vast majority of current evidence regarding the PMN derives from murine models. Because PMN formation occurs during the early stage preceding the establishment of metastatic foci, and because the deep location of the liver renders continuous dynamic sampling difficult, direct observation of PMN formation in humans is practically infeasible; clinical studies can typically only examine specimens from established metastatic lesions, making it difficult to capture the dynamic changes of PMN formation. This limitation hinders the clinical translation of PMN-targeted therapeutic candidates, as the appropriate timing and efficacy of interventional strategies shown to be effective in murine models remain difficult to verify in humans. Consequently, future efforts are urgently needed to develop surrogate markers and detection methods capable of reflecting the process of human PMN formation, in order to advance the clinical translation of PMN-targeted therapies.

### 2.3 Circulating tumor cells and epithelial-mesenchymal transition

If the formation of the PMN serves to create a microenvironment permissive for metastasis, the dissemination of circulating tumor cells (CTCs) acts as the pivotal executor of metastatic colonization. CTCs are tumor cells that have detached from the primary neoplasm and entered the peripheral blood circulation. Although the overwhelming majority of CTCs are eliminated in the circulation by immune surveillance or undergo apoptosis due to hemodynamic shear stress, a small fraction with specialized biological properties can survive and successfully colonize distant organs (59). These surviving CTCs exhibit heterogeneous states, including epithelial, hybrid (partial), and mesenchymal phenotypes. Although abundant *in vitro* and animal model studies suggest that EMT can endow tumor cells with invasive, migratory, and anti-apoptotic capacities and is associated with the acquisition of cancer stemness and therapeutic resistance, EMT is not a universal prerequisite for intrahepatic colonization; the detection of EMT markers alone does not establish functional causality, and metastatic dissemination can also proceed through collective migration and intravascular invasion without a complete EMT.

#### 2.3.1 Molecular regulation of EMT

EMT is a biological process wherein epithelial cells lose their polarity, adhesion, and epithelial markers while concomitantly gaining mesenchymal characteristics and enhanced motility (16). In CRC, EMT not only equips tumor cells with the capacity for invasion and metastasis but is also closely associated with the acquisition of cancer stemness and therapeutic resistance.

The activation of EMT in CRC is coordinately regulated by multiple signaling pathways. The TGF-β/SMAD pathway constitutes one of the most canonical inducers of EMT; the upregulation of urea transporter proteins stabilizes TβRII, thereby potentiating TGF-β signaling and inducing EMT through the phosphorylation and nuclear translocation of SMAD2/3 (60). The WNT/β-catenin pathway, which is the most frequently aberrantly activated oncogenic pathway in CRC (chiefly driven by APC mutations), also contributes to EMT; overexpression of C6orf15, a novel uncharacterized protein, activates this pathway to induce EMT and hepatic metastasis (61). Additionally, the MAPK/ERK and PI3K/AKT/mTOR pathways are involved in EMT regulation; loss of the protein phosphatase 2 scaffold subunit Aβ activates the former to induce EMT (62), whereas overexpression of arylamine N-acetyltransferase 1 (NAT1) blocks the latter, inhibiting EMT and VEGF expression and thereby decelerating the progression of colorectal cancer liver metastasis (63).

Non-coding RNAs participate equally in EMT regulation. The circular RNA circ0020134 acts as a molecular sponge for miRNA-183-5p, upregulating PFN2 and activating the TGF-β/SMAD pathway to drive EMT (64). The long non-coding RNA SNHG16 forms a positive feedback loop with the YAP1/TEAD1 complex: SNHG16 relieves the repression of YAP1 by miR-195-5p, while the YAP1/TEAD1 complex in turn positively regulates SNHG16 transcription, thereby promoting the EMT of CTCs and CRC liver metastasis (17).

#### 2.3.2 Survival mechanisms and intercellular crosstalk of CTCs

Upon entering the circulation, CTCs confront a multifaceted survival challenge, including anoikis, fluid shear stress, and immune surveillance. To survive, CTCs have evolved sophisticated adaptive mechanisms and intercellular crosstalk strategies.

The interaction between CTCs and platelets represents a core strategy for immune evasion. CD47 expressed on the surface of CTCs interacts with platelets, leading to platelet activation and aggregation, which forms a ‘platelet cloak.’ This cloak not only provides physical protection against hemodynamic shear forces but also inhibits anoikis through YAP1 signaling (18). Furthermore, the platelet cloak impedes the recognition and killing of CTCs by NK cells. Activated platelets also secrete TGF-β, which helps maintain the EMT state of CTCs (65).

The interaction between CTCs and neutrophils also plays a significant metastasis-promoting role. It has been demonstrated (19) that neutrophils can bind to CTCs via VCAM-1, transfer to distant organs, and enhance the proliferative capacity of CTCs through cell cycle regulation. In CRC, NETs have been found to capture CTCs in hepatic sinusoids and induce the expression of IL-8 in tumor cells; elevated IL-8 levels further activate neutrophils to produce more NETs, forming a malignant positive feedback loop that drives CRLM progression (20).

CTCs can also disseminate through the circulation as multicellular clusters. In breast cancer, although these clusters are present at substantially lower frequencies than individual CTCs, their metastatic potential is elevated by several orders of magnitude-reportedly up to 23- to 50-fold (66). Cells within CTC clusters exhibit enhanced stemness features and an EMT phenotype, and the heterogeneous cellular composition of these clusters-which can include platelets, neutrophils, and fibroblasts-provides complementary support functions (67). Gkountela et al. (68) discovered that the promoter regions of stemness-associated transcription factors, such as Octamer-binding transcription factor 4 (Oct4) and Homeobox protein NANOG, are hypomethylated in CTC clusters, leading to their elevated expression and enhanced cellular self-renewal capacity. Although CTC clusters carry important prognostic value, their clinical detection rate remains low and standardized detection protocols are lacking, limiting their implementation as a routine clinical biomarker.

Collectively, the above studies indicate that CTCs can acquire invasive and anti-apoptotic properties through phenotypic plasticity-including EMT, hybrid EMT states, and dynamic interactions with the microenvironment-and rely on mechanisms such as platelet cloaking, neutrophil escort, and cluster-based dissemination to survive within the circulation. Once these ‘seeds’ reach the hepatic microenvironment, which has been preconditioned by the PMN to possess profound immunosuppressive characteristics, they may initiate the processes of colonization and expansion; however, direct evidence for the majority of these mechanisms in human CRLM remains limited, and the relevant conclusions derive principally from model systems.

### 2.4 Immune cell and cytokine networks within the tumor microenvironment

Once CRLM is established, the intricate network of immune cells and cytokines within the TME exerts a decisive influence on tumor growth, immune escape, and therapeutic response (**Figure 3**). The TME of CRLM is distinguished from that of other organ metastases by its profound immunosuppression and T cell exclusion, features that are considered closely associated with its low responsiveness to immunotherapy.

**Figure 3:**
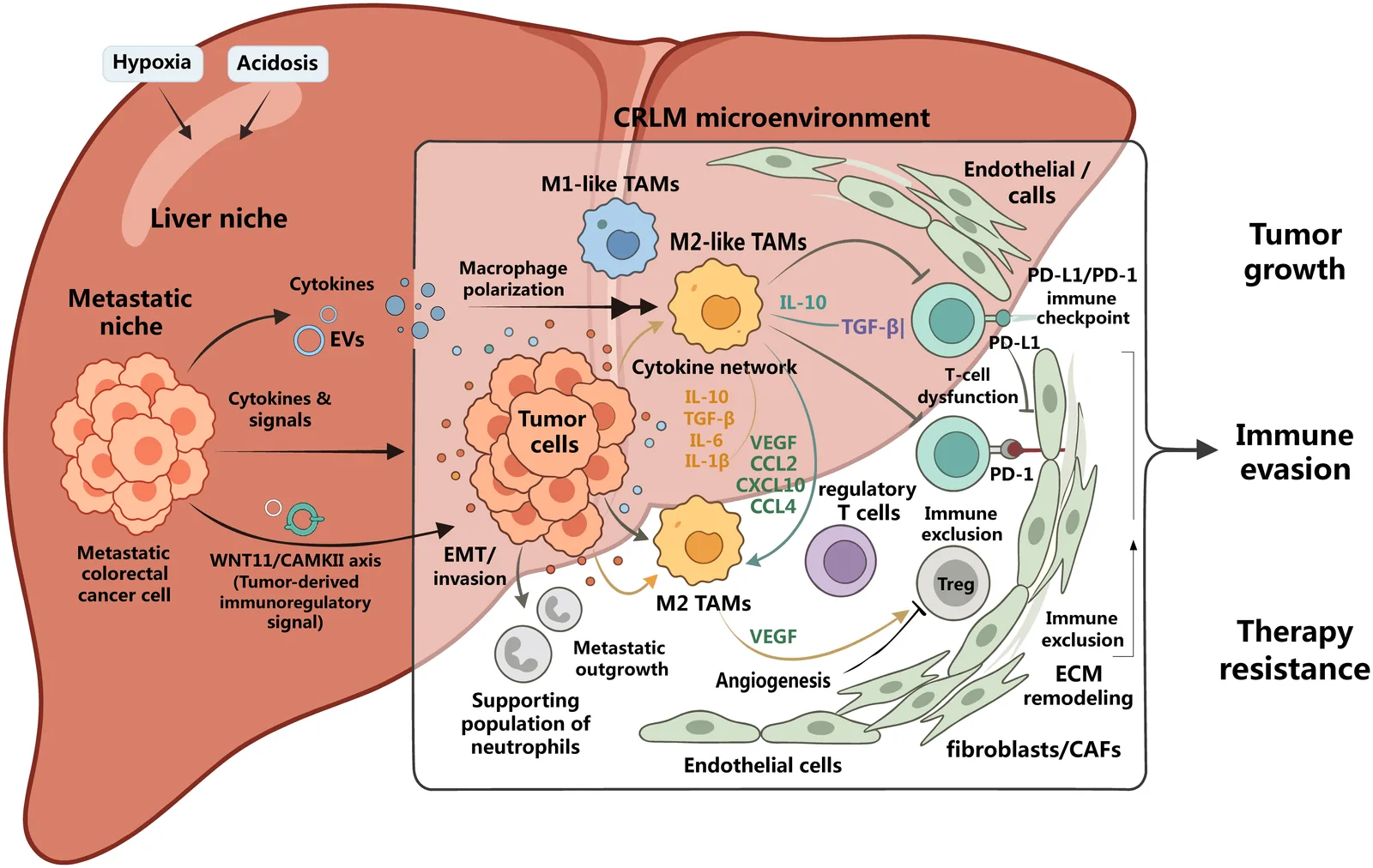
The multicellular regulatory network within the tumor microenvironment of colorectal cancer liver metastasis. Tumor-derived cytokines orchestrate the polarization of macrophages toward a pro-tumorigenic M2 phenotype, whereupon M2-polarized tumor-associated macrophages (TAMs), regulatory T cells (Tregs), and cancer-associated fibroblasts (CAFs) cooperatively mediate angiogenesis, extracellular matrix remodeling, and the activation of the PD-L1/PD-1 signaling axis, thereby collectively sustaining a profoundly immunosuppressive microenvironment that facilitates tumor proliferation, immune evasion, and therapeutic resistance. Evidence level: The analyses of the spatial co-localization and phenotypic characteristics of macrophages, Tregs and CAFs are based on single-cell transcriptomics and spatial transcriptomics data from human colorectal liver metastasis tissues and mouse models. Causal direction: The conclusion that these cell populations collectively promote tumor proliferation, immune escape and therapeutic resistance remains at the stage of associative or mechanistic inference, while direct functional validation in human subjects is still insufficient.

#### 2.4.1 Polarization and immunosuppression of tumor-associated macrophages

Tumor-associated macrophages (TAMs) are among the most abundant immune cell populations in the CRLM TME, exhibiting remarkable phenotypic and functional plasticity. Under the stimulation of signals such as IFN-γ and LPS, TAMs can differentiate into a pro-inflammatory, anti-tumor M1 phenotype; conversely, driven by signals including IL-4, IL-10, and TGF-β, they polarize toward an anti-inflammatory, pro-tumor M2 phenotype (69). It should be emphasized, however, that this binary classification framework constitutes merely a simplified, pedagogically oriented model that cannot fully capture the continuous, overlapping, and microenvironment-dependent activation states of myeloid cells in human tumors. In the hepatic context, TAMs can be categorized by ontogeny into two populations: embryo-derived resident Kupffer cells and macrophages differentiated from circulating monocytes, which exhibit substantial heterogeneity in surface markers and biological functions and therefore cannot be discussed homogenously. Accordingly, this review retains the M1/M2 terminology only when describing original studies that initially adopted this classification; when discussing findings derived from human single-cell transcriptomic or spatial transcriptomic analyses, macrophage subsets defined by marker or functional characteristics (e.g., SPP1-high macrophages, TREM2-positive macrophages) are employed, avoiding the indiscriminate assignment of all TAMs possessing both immunosuppressive and pro-tumorigenic properties to a single “M2” category. In CRLM, multiple microenvironmental signals within the TME-including hypoxia, lactate accumulation, and tumor-derived exosomes-can induce TAMs to acquire pro-tumorigenic functional phenotypes characterized by immunosuppression, pro-angiogenesis, and pro-fibrosis; these biological effects extend far beyond a simple “M2 polarization” process and collectively construct a profoundly immunosuppressive tumor microenvironment.

The molecular mechanisms driving macrophages toward an immunosuppressive pro-tumorigenic phenotype involve multiple signaling pathways. It has been demonstrated that CRC exosomal miR-934 promotes the pro-tumorigenic function of macrophages through activation of the PTEN/PI3K/AKT pathway (42). Li et al. (70) found that cathepsin K (CTSK), produced by tumor cells under the stimulation of the gut microbiota, binds to TLR4 on the macrophage surface and promotes macrophage differentiation toward an immunosuppressive state through an mTOR-dependent mechanism, subsequently stimulating the secretion of IL-10 and IL-17, which further enhance CRC invasion and metastasis via the NF-κB pathway. Furthermore, the loss of STING signaling attenuates NLRP3-mediated production of IL-18 and IL-1β, impairing the anti-tumor activity of NK cells and thereby creating favorable conditions for CRLM (71).

At the spatial organizational level, the application of single-cell and spatial transcriptomic technologies has revealed highly ordered interactions between TAMs and stromal cells in CRLM. Qi et al. (72) discovered that SPP1-high TAMs are specifically enriched in the vicinity of FAP+ CAFs at the invasive margin, effectively preventing T cell infiltration into the tumor nest while concurrently upregulating immune checkpoint ligand expression and reducing antigen-presentation capacity, which is closely correlated with poor clinical prognosis.

#### 2.4.2 T cell dysfunction and immune exclusion

CD8+ cytotoxic T lymphocytes (CTLs) are the core effector cells of adaptive anti-tumor immunity, capable of directly killing tumor cells through the release of perforin and granzymes. However, within CRLM, T cells are subjected to multiple suppressive mechanisms leading to profound functional impairment, a state termed T cell exhaustion.

First, physical barriers limit the contact between T cells and tumor cells. SPP1+ TAMs and FAP+ CAFs construct a physical barrier around the tumor by secreting collagen-rich ECM, resulting in the entrapment of T cells at the tumor periphery and hindering their infiltration into the parenchyma (72). In this process, TGF-β signaling plays a critical role: TGF-β not only directly inhibits the killing function of cytotoxic T lymphocytes (CTLs) but also stimulates CAFs to produce a dense ECM, further exacerbating the physical exclusion of T cells (73).

Second, the persistent activation of immune checkpoint pathways leads to T cell functional exhaustion. Tumor cells and immunosuppressive cells in CRLM highly express PD-L1, while T cells upregulate multiple inhibitory receptors, including programmed death receptor-1 (PD-1), cytotoxic T lymphocyte antigen-4 (CTLA-4), T cell immunoglobulin and mucin domain-containing protein 3 (TIM-3), lymphocyte activation gene-3 (LAG-3), and T cell immunoreceptor with Ig and ITIM domains (TIGIT). Chronic activation of the PD-1/PD-L1 signaling axis induces T cells to enter a dysfunctional state characterized by reduced production of effector cytokines (IFN-γ, TNF-α, IL-2), decreased proliferative capacity, and increased apoptosis (74).

Third, unique immunotolerant mechanisms within the liver systemically eliminate activated T cells. Yu et al. (28) demonstrated in a murine model that FasL+CD11b+F4/80+ macrophages within liver metastases induce the apoptosis of Fas+CD8+ T cells through Fas-FasL interactions, resulting in a systemic reduction of CD8+ T cell numbers both locally within metastatic lesions and in the peripheral circulation. This preclinical evidence provides important clues for explaining the primary resistance of patients with liver metastases to immune checkpoint inhibitors and suggests the hypothesis that liver metastases may function as drivers of systemic immune resistance. However, direct functional validation in humans is currently lacking; the extent to which the same mechanism operates in human CRLM and its quantitative impact on immunotherapy efficacy await confirmation through prospective clinical studies.

Finally, Tregs are highly enriched in the CRLM TME and exert potent immunosuppressive functions. Tregs suppress anti-tumor immunity through a multitude of mechanisms, including the production of inhibitory cytokines (IL-10, TGF-β, IL-35), the consumption of IL-2, the downregulation of co-stimulatory molecules on antigen-presenting cells (APCs) via CTLA-4, and the direct killing of effector T cells (75). Lee et al. (76) demonstrated that liver metastases can induce CTLA-4-high Treg-dependent immunosuppression that renders anti-PD-1 monotherapy ineffective unless combined with CTLA-4 blockade to abrogate or overcome this Treg-mediated suppression.

#### 2.4.3 The pro-metastatic function of the cytokine network

Within the CRLM TME, the cytokine network constitutes a central signaling system that drives the metastatic cascade. IL-6 is among the most extensively studied pro-metastatic cytokines. In CRC, IL-6 induces EMT by activating the JAK2/STAT3 signaling pathway, significantly enhancing the migratory, invasive, and distant metastatic capacities of tumor cells (77). More critically, IL-6 drives an immunosuppressive positive feedback loop: activated STAT3 signaling promotes the production of IL-6 by tumor cells, which in turn recruits MDSCs to the hepatic PMN, and the recruited MDSCs further produce IL-6 to reinforce this loop (47).

IL-8 also plays a critical pro-metastatic role in CRLM. By binding to its receptors CXCR1 and CXCR2, IL-8 promotes CRC cell proliferation, induces angiogenesis and EMT, and is considered one of the core chemokines driving CRLM progression (78). As previously described, IL-8 participates in a NET-mediated metastatic positive feedback loop: NETs capture CTCs and induce IL-8 expression, and elevated IL-8 further activates neutrophils to produce more NETs (20). Furthermore, IL-8 can induce the expression of LSECtin protein through the AKT signaling pathway, which is closely associated with the distant metastatic capacity of CRC cells (79).

CCL2 primarily mediates the chemotactic recruitment of monocytes and macrophages via the CCR2 receptor. In CRLM, CCL2 secreted by CRC cells mobilizes CD11b+/Gr-1mid/CCR2+ monocytes/macrophages from the bone marrow to the hepatic metastatic sites through CCR2 activation, accelerating disease progression via pro-angiogenic effects (80). Genome-wide association analysis further indicates that polymorphisms at the monocyte chemoattractant protein-1 (MCP-1) locus are significantly associated with differential expression in CRLM patients, suggesting that genetic variation in MCP-1 may influence individual susceptibility to hepatic metastasis (81).

CXCL5 activates the AKT/NF-κB/FOXD1/VEGF-A signaling pathway through the CXCR2 axis to promote tumor angiogenesis (82) and enhances tumor cell migratory and invasive capacities through the ERK/Elk-1/Snail and AKT/GSK3β/β-catenin/MMP7 pathways, respectively (83). Serum CXCL5 levels are significantly elevated in CRC patients and are closely correlated with metastatic risk and adverse prognosis (84).

Within the TME, pro-tumorigenic macrophages, dysfunctional CD8+ T cells, Tregs, and the cytokine network comprising IL-6/IL-8/CCL2 collectively construct an immunosuppressive tumor microenvironment that is deeply coupled with intracellular signaling pathways and metabolic reprogramming, jointly determining tumor biological behavior and therapeutic resistance.

### 2.5 The synergistic regulatory network of core signaling pathways

The initiation and progression of CRLM are not the consequence of a single isolated signaling pathway but rather the product of synergistic interactions among multiple core pathways that form a regulatory network. Among these, the PI3K/AKT, WNT/β-catenin, and TGF-β/SMAD pathways constitute the most critical signaling axes in CRLM, coordinately driving EMT, immune evasion, and metabolic reprogramming through direct or indirect crosstalk.

The TGF-β/SMAD pathway acts as a central inducer of EMT, and it can cross-activate the PI3K/AKT pathway through SMAD-independent mechanisms, enhancing cell survival signals and metabolic adaptation, thereby providing anti-apoptotic protection to CRC tumor cells undergoing EMT and helping them overcome the threat of anoikis during the metastatic cascade (85). More than 80% of sporadic CRCs harbor mutations in APC or CTNNB1, leading to aberrant activation of the WNT/β-catenin pathway, which drives the expression of EMT- and stemness-related genes; this pathway also engages in positive feedback crosstalk with the PI3K/AKT pathway, whereby AKT-mediated phosphorylation and inactivation of GSK-3β inhibits β-catenin ubiquitination and degradation, further enhancing WNT signaling and functionally coupling metabolic signals with developmental signals to coordinately propel metastatic progression (86).

The three pathways also act in concert at the level of TME remodeling. TGF-β drives matrix restructuring and immunosuppression by inducing CAF activation (73); PI3K/AKT promotes tumor angiogenesis by regulating the expression of VEGF and other angiogenic factors; and WNT signaling participates in the colonization process of metastatic foci by modulating the adhesion between LSECs and tumor cells (87).

Furthermore, these three pathways form a synergistic regulatory network in hepatic metastasis, maintaining the EMT and stemness characteristics of the ‘seed’ while simultaneously promoting immune escape through remodeling of the ‘soil.’ The control of downstream effector terminals by these pathways is largely achieved through metabolic and epigenetic mechanisms.

### 2.6 Metabolic reprogramming and the emerging role of lactylation

Metabolic reprogramming is regarded as a core hallmark of cancer (88). CRC cells universally exhibit enhanced aerobic glycolysis, known as the Warburg effect, wherein they preferentially convert glucose to lactate even under conditions of ample oxygen. This metabolic shift holds dual significance: on the one hand, rapid glycolysis provides essential biosynthetic precursors (such as nucleotides, amino acids, and lipids) for tumor cell proliferation; on the other hand, the accumulation of high concentrations of lactate within the TME drives TME acidification, immunosuppression, and a newly discovered type of protein post-translational modification-lactylation (Kla).

#### 2.6.1 Glycolytic reprogramming promotes CRLM

CRC cells reprogram their glucose metabolism through diverse mechanisms to meet the energetic and biosynthetic demands of the intrahepatic metastatic niche. Zhao et al. (89) identified 444 lactylation sites on 637 proteins in CRC cells, including the key glycolytic enzyme phosphofructokinase (PFKP). The lactylation of PFKP at the K688 site reduces its enzymatic activity, forming a negative feedback control on glycolytic flux to prevent cytotoxicity from excessive lactate accumulation. This finding reveals a functional significance of lactylation as a metabolic homeostatic regulatory mechanism.

CKLF-like MARVEL transmembrane domain-containing 6 (CMTM6) has been found to maintain the Warburg effect in CRC by regulating the membrane localization of glucose transporter 1 (GLUT1). The loss of CMTM6 leads to the lysosomal degradation of GLUT1, inhibits glucose uptake and glycolysis, and potently suppresses liver metastasis in mouse models (35). Ketohexokinase-A (KHK-A), on the other hand, phosphorylates pyruvate kinase M2 (PKM2) at the Ser37 site, inhibiting its tetramer formation and pyruvate kinase activity, and enhancing PKM2 nuclear translocation, thereby exacerbating the Warburg effect and EMT and driving fructose-dependent CRLM (36). Furthermore, the circular RNA circNOLC1 interacts with zinc-α2-glycoprotein 1 (AZGP1) to activate the mTOR/SREBP1 signaling axis, upregulating glucose-6-phosphate dehydrogenase (G6PD) expression and enhancing the metabolic flux of the pentose phosphate pathway, which provides ribose-5-phosphate for nucleotide synthesis and facilitates CRLM progression (90).

#### 2.6.2 The pro-metastatic role of lipid metabolism reprogramming

Beyond glucose metabolism, the reprogramming of lipid metabolism, particularly cholesterol metabolism, significantly contributes to the malignant progression of CRLM. As the central organ for cholesterol synthesis, the liver provides a unique metabolic adaptation opportunity for CRLM. Taniguchi et al. (37), using a murine model of colon cancer liver metastasis, isolated a subpopulation of tumor cells enriched in fatty acids and cholesterol and found that these cells undergo apoptosis upon *in vitro* treatment with cholesterol synthesis inhibitors, indicating that CRC cells with high metastatic potential exhibit cholesterol-dependent growth characteristics.

Zhang et al. (38) further demonstrated that hepatocyte-derived hepatocyte growth factor (HGF) activates the c-Met/PI3K/AKT/mTOR signaling axis in CRC cells, which subsequently triggers the sterol regulatory element-binding protein 2 (SREBP2)-dependent cholesterol biosynthesis pathway. This mechanism suggests that metastatic CRC cells can exploit the abundant HGF in the liver environment to trans-activate their own cholesterol synthesis program, meeting the enormous demand for membrane components and signaling molecules during rapid metastatic expansion. Consequently, strategies targeting the cholesterol synthesis pathway, such as statins (HMG-CoA reductase inhibitors), may serve as effective adjuncts in the treatment of CRLM.

#### 2.6.3 Lactylation: an emerging link between metabolism and epigenetics

Lactate has long been regarded as a mere metabolic waste product; however, a landmark study published in 2019 by Zhang et al. (33) revealed that lactate can directly regulate gene transcription by covalently modifying histone lysine residues to form lactylation (Kla), thereby establishing a direct link between metabolic status and epigenetic regulation. Although lactylation has been associated with tumor proliferation, metastasis, immune escape, and chemoresistance, the majority of evidence derives from colorectal cancer cell lines and murine models, while direct data from human CRLM remain limited; consequently, this review regards lactylation as an emerging hypothesis rather than an established mechanism.

In the promotion of proliferation, Chen et al. (91) found that lactate accumulation not only activates the transcription of NOP2/Sun RNA methyltransferase family member 2 (NSUN2) by promoting H3K18 lactylation but also directly induces NSUN2 lactylation at the K388 site, thereby enhancing m5C RNA methylation modification and promoting CRC cell proliferation. At the translational regulation level, Xie et al. (92) revealed a key function of lysine acetyltransferase 8 (KAT8) as a lactyltransferase in CRC: KAT8 catalyzes the lactylation of the eukaryotic translation elongation factor eEF1A2 at the K408 site, a modification that enhances the interaction of eEF1A2 with ribosomes and significantly increases the rate of protein translational elongation, meeting the enormous demand for protein synthesis in rapidly proliferating CRC cells.

In the promotion of metastasis, lactylation drives CRC invasion and distant dissemination through multiple mechanisms. G protein-coupled receptor 37 (GPR37) maintains high levels of H3K18la by activating the Hippo pathway, which in turn induces the secretion of CXCL1 and CXCL5, recruiting neutrophils to the liver and potentially assisting PMN construction via NETs (93). Regarding immune escape, Tong et al. (34) recently identified GCN5 (KAT2A) as a critical lactyltransferase for PD-L1: GCN5-catalyzed PD-L1 lactylation hinders its lysosomal degradation pathway, significantly increasing the stability and abundance of PD-L1 on the tumor cell surface. This ‘GCN5–PD-L1 Kla’ axis not only enhances tumor cell resistance to T cell killing but also provides a metabolic–epigenetic mechanistic explanation for how a serine/glycine-restricted diet influences the efficacy of immunotherapy.

In the context of chemoresistance, lactylation promotes resistance to standard chemotherapeutic agents (5-fluorouracil and oxaliplatin) in CRC. A breakthrough discovery by Chen et al. (94) elucidated that p300/CBP-catalyzed lactylation of the meiotic recombination 11 (MRE11) protein at the K673 site significantly enhances the DNA binding affinity of MRE11, accelerating homologous recombination (HR) repair processes, thereby enabling CRC cells to rapidly repair DNA damage induced by platinum-based drugs or poly (ADP-ribose) polymerase (PARP) inhibitors. Furthermore, Deng et al. (95) found that CRC stem cells upregulate the expression of the glutamate-cysteine ligase catalytic subunit (GCLC) through histone H4K12 lactylation, mediated collaboratively by lactate dehydrogenase A (LDHA) and p300, inhibiting ferroptosis and conferring resistance to oxaliplatin.

It is essential to emphasize that the function of lactylation in CRC is highly context-dependent. Although the majority of evidence supports a pro-tumorigenic role for lactylation, it may also exert opposite functions in specific contexts. For instance, the PFKP K688 lactylation-mediated negative feedback on glycolysis may restrain tumor growth in the early stages, while in other non-CRC models, lactylation can also stabilize or activate tumor suppressors such as wild-type p53 (89, 96). Therefore, the net effect of lactylation depends on the functional positioning of its modification substrate (within oncogenic versus tumor-suppressive pathways), its subcellular localization (nuclear histone lactylation primarily regulates transcription, whereas cytoplasmic non-histone lactylation fine-tunes metabolic flux), and the stage of disease. However, most current lactylation studies have focused primarily on tumor cells themselves, and direct *in vivo* evidence for protein lactylation in immune cells (such as macrophages and T cells) within CRLM remains limited. Future investigations will need to resolve the spatiotemporal dynamics of lactylation at single-cell resolution to avoid unintended consequences arising from non-selective therapeutic inhibition.

The glycolytic and lipid metabolic reprogramming of CRC liver metastases not only provides biosynthetic substrates but also mediates protein lactylation through the accumulation of metabolites such as lactate, directly converting metabolic status into epigenetic instructions. These ‘environmental modifiers’ can simultaneously act on tumor cells and immune cells, profoundly influencing proliferation, metastasis, immune escape, and chemoresistance. Beyond this, another class of important environmental modifiers-the gut microbiota and its metabolic products-also participates in the initiation and progression of colorectal cancer liver metastasis.

### 2.7 The gut microbiota as an ‘environmental modifier’

Unlike most solid tumors, the initiation and progression of CRC are inextricably linked to its associated intestinal ecosystem. The gut microbiota-comprising trillions of bacteria, fungi, and viruses-is profoundly involved in the regulation of CRLM through interactions with the host immune system, long-distance signaling by metabolic products, and the direct carcinogenic effects of specific pathogenic bacteria (97). The discovery of this ‘microbiota–metabolism–epigenetics’ axis introduces a completely new dimension to the understanding of CRLM.

#### 2.7.1 Gut dysbiosis and its relationship with CRLM

The gut microbial composition of CRC patients differs significantly from that of healthy individuals, characterized by an enrichment of specific pathogenic bacteria (e.g., Fusobacterium nucleatum, enterotoxigenic Bacteroides fragilis, Escherichia coli) and a reduction of beneficial commensals (e.g., butyrate-producing Faecalibacterium prausnitzii). This dysbiosis not only contributes to the initiation and local progression of CRC but is also closely related to the development of hepatic metastasis.

Fusobacterium nucleatum is the most extensively studied pro-tumorigenic bacterium in CRC. Clinical retrospective studies have found that the colonization of F. nucleatum in CRLM tissues is closely associated with systemic inflammatory responses and the remodeling of the tumor immune microenvironment (30). Mechanistically, F. nucleatum-positive tumor tissues display significantly reduced CD8+ T cell infiltration and an accumulation of MDSCs, suggesting that this bacterium may promote tumor immune escape by inducing an immunosuppressive state (98). Moreover, F. nucleatum enhances chemoresistance in tumor cells by activating the TLR4/MyD88 signaling pathway and can influence the expression of IL-8 by regulating miR-5692a, exacerbating intestinal epithelial inflammation to accelerate CRLM (99).

Escherichia coli participates in CRLM through distinct mechanisms. Studies have identified that the virulence factor VirF of E. coli C17 can specifically disrupt the gut vascular barrier (GVB), and the impairment of the GVB is a necessary prerequisite for bacterial translocation to the liver and the subsequent recruitment of inflammatory cells and acceleration of tumor metastasis (31). Gu et al. (32) recently discovered that lactate produced by the intra-tumoral microbiota induces lactylation of the retinoic acid-inducible gene I (RIG-I) protein at the K852 site in macrophages, inhibiting the NF-κB signaling pathway, promoting M2 macrophage polarization and driving liver metastasis-a finding that directly connects the microbiota, metabolism, and epigenetic regulation in series.

#### 2.7.2 Microbial metabolites and liver metastasis

Metabolites produced by the gut microbiota, particularly short-chain fatty acids (SCFAs) and secondary bile acids, serve as important chemical messengers linking the gut ecosystem to hepatic biology. SCFAs, which primarily include butyrate, propionate, and acetate, are generated by bacterial fermentation of dietary fiber and possess protective functions in inhibiting tumor growth and maintaining intestinal barrier integrity (100). Butyrate modulates CD4+ T cell differentiation by binding to the GPR109a receptor on the surface of dendritic cells and macrophages, increasing the frequency of Tregs and IL-10-secreting CD4+ T cells while reducing the Th17 cell population, thereby suppressing chronic inflammation-driven tumor progression (101).

In contrast to the protective role of SCFAs, secondary bile acids (such as deoxycholic acid and lithocholic acid) exhibit pro-carcinogenic activity. Dysbiosis in CRC patients is often accompanied by an increased abundance of bile acid-metabolizing bacteria (e.g., Clostridium species). Lithocholic acid has been found to stimulate IL-8 secretion from CRC cells and promote tumor progression and metastasis through the activation of the ERK1/2 MAPK pathway (102). In animal models, the administration of broad-spectrum antibiotics to control gut microbial abundance significantly reduces serum levels of secondary bile acids and the number of hepatic metastatic foci, confirming the potential value of targeted modulation of the gut microbiota in intervening in bile acid-mediated CRLM (103).

#### 2.7.3 The gut vascular barrier

The gut vascular barrier (GVB) is a structure that has recently been recognized as playing a critical role in CRLM. Spadoni et al. (104) first described the existence and function of the GVB in Science: analogous to the blood-brain barrier, the GVB effectively prevents the entry of molecules with a molecular weight of ≥70 Da from the intestinal lumen into the portal venous system, maintaining the homeostasis of the intestinal internal environment. In CRC, the disruption of the GVB is a critical step in the development of hepatic metastasis. Clinical observations have revealed that plasma levels of PV-1, a marker of GVB injury, are significantly elevated in CRC patients, and the bacterial load within liver metastases increases with disease progression (31). In experimental interventional studies, hyperlipidemic CRC mice treated with Dachaihu decoction, a traditional Chinese herbal formula, exhibited attenuated GVB damage and a reduced rate of liver metastasis, suggesting that preserving GVB integrity may represent an effective strategy for the prevention of CRLM (105).

The multi-layered regulatory mechanisms described above-from the ‘seed’ to the ‘soil’ and further to the ‘environmental modifiers’-systematically elucidate the complex biological process by which tumor cells detach from the primary site, survive in the circulation, colonize the liver, and ultimately form metastatic foci. They also clarify the critical regulatory steps by which the hepatic microenvironment undergoes malignant remodeling to support metastatic outgrowth, providing a rich repertoire of molecular candidates and a solid theoretical foundation for the early detection, prognostic stratification, formulation of diagnostic strategies, development of therapeutic modalities, refinement of prognostic assessment systems, and identification of therapeutic targets in CRLM, while uncovering a series of candidate targets with clinical translational potential. Furthermore, when extrapolating microbiota-related mechanisms to patients with CRLM, several major confounding factors must be acknowledged. First, the compositions of fecal, intestinal mucosal, tumor tissue, and intra-tumoral microbiota are not equivalent; studies of intra-tumoral/tissue microbiota are susceptible to sampling contamination and low-biomass issues, limiting the reproducibility of results. Second, antibiotic use, dietary structure, bowel preparation, and concomitant medications can significantly alter microbial composition, yet most retrospective studies have not standardized the documentation of these factors. Third, microbial composition exhibits substantial geographic, dietary, and population differences, making associations derived from a single center or region difficult to extrapolate. Fourth, most current evidence on the relationship between the microbiota and CRLM consists of cross-sectional associations or causal evidence obtained in animal models; there remains a lack of interventional evidence demonstrating that baseline microbial characteristics prospectively predict subsequent liver metastasis and that targeted microbiota modulation alters metastatic outcomes in humans. Accordingly, this review regards the ‘microbiota–metabolism–epigenetics’ axis as an emerging direction for future research; the relevant conclusions still require validation through larger population-based studies and targeted interventional trials, and the specific regulatory mechanisms await further in-depth investigation.

## 3 Diagnosis and biomarkers of colorectal cancer liver metastasis

The early diagnosis and precise monitoring of CRLM are paramount to improving patient outcomes. Current clinical diagnostic strategies integrate two major pillars: serum tumor biomarkers and imaging evaluations, while emerging liquid biopsy technologies and molecular markers offer promise for earlier and more accurate prediction.

### 3.1 Traditional biomarkers and imaging diagnosis

Serum carcinoembryonic antigen (CEA) is the most commonly used first-line tumor marker in CRC patients, and its value in treatment monitoring and recurrence alerting is broadly recognized. Carbohydrate antigen 19-9 (CA19-9) serves a complementary diagnostic role in a subset of CRLM patients who do not express or express low levels of CEA (106). However, the sensitivity and specificity of CEA and CA19–9 are not ideal, and reliance on these markers alone for the early screening and diagnosis of CRLM is significantly limited.

Imaging examinations are the core modalities for the diagnosis and assessment of CRLM. Conventional hepatic ultrasonography is simple and accessible but has limited sensitivity and specificity. Contrast-enhanced abdominal CT is currently the most commonly utilized method, capable of providing critical information on the location, size, number, and vascular relationships of metastatic lesions. When ultrasound or CT findings are inconclusive, hepatic MRI, owing to its superior soft-tissue resolution, is the preferred modality for further confirmation (107). PET-CT, while not a routine standard examination for CRLM, possesses unique value in specific clinical scenarios, such as assessing the extent of extrahepatic disease or distinguishing viable tumor from post-treatment changes. A needle biopsy of hepatic metastases is generally not routinely recommended, due to the risk of needle-tract seeding and hemorrhage-particularly for tumors adjacent to great vessels-and because non-invasive imaging techniques can provide sufficient diagnostic information (108).

For post-operative surveillance, current guidelines recommend monitoring CEA and CA19–9 levels every 3–6 months for the first 2 years, then every 6 months for the subsequent 3 years, and annually after 5 years (109). Imaging follow-up is recommended every 3 months with contrast-enhanced abdominal/pelvic CT or enhanced liver MRI (preferably using a liver-specific contrast agent) for the first 2 years post-surgery, followed by consistent imaging modalities every 6–12 months for the next 5 years (110).

### 3.2 Advances in molecular biomarkers

Beyond traditional clinicopathological parameters, a variety of molecular markers have demonstrated significant predictive value for the prognosis and treatment response of CRLM.

The mutational status of RAS genes (KRAS and NRAS) is one of the most important predictive molecular markers for CRLM patients, directly influencing the efficacy of anti-EGFR targeted therapy. KRAS is mutated in approximately 40% of CRCs, and patients harboring RAS mutations exhibit primary resistance to anti-EGFR monoclonal antibodies, such as cetuximab and panitumumab (111). Consequently, testing for RAS mutations is recommended for all CRLM patients to guide treatment decisions. BRAF mutations, particularly BRAF V600E, constitute a critical prognostic biomarker, as patients with BRAF-mutated CRLM have a significantly poorer prognosis (112). Furthermore, the detection of MSI/MMR status is not only crucial for screening for Lynch syndrome but also directly determines the applicability of immunotherapy: patients with MSI-H/dMMR tumors have a substantially higher response rate to ICIs (113).

Liquid biopsy technologies-particularly the detection of CTCs and circulating tumor DNA (ctDNA)-have undergone rapid development in recent years. In CRLM, the enumeration and molecular characterization of CTCs are regarded as a ‘liquid window’ reflecting tumor burden and metastatic potential. Cayrefourcq et al. (15) identified a unique transcriptional signature in CTCs isolated post-treatment, including the upregulation of genes in the mTOR and PI3K/AKT signaling pathways and the expression of stemness and plasticity markers, which are closely associated with treatment selection and acquired drug resistance. ctDNA detection monitors tumor dynamics and molecular evolution by analyzing tumor-derived free DNA in plasma. Studies have shown that the detection of RAS mutations in ctDNA can predict acquired resistance to anti-EGFR therapy weeks to months before radiological progression, providing a ‘molecular warning’ that allows for timely adjustment of treatment regimens (114). At the methodological level of longitudinal molecular monitoring, studies have demonstrated feasible frameworks for tracking tumor dynamics and the evolution of resistant clones through serial liquid biopsies (115, 116); it should be emphasized that these methods do not constitute direct clinical evidence in CRLM, and their applicability to CRLM requires dedicated validation.

Of note, tumor-derived exosomes are receiving increasing attention as potential targets for liquid biopsy. The miRNA, protein, and lipid cargo of exosomes reflects the molecular characteristics of the parental tumor, and their high stability in body fluids renders them valuable for biomarker development. For instance, the levels of serum exosomal miR-934 and miR-181a-5p are closely correlated with CRC liver metastasis and adverse prognosis (25, 42). Integrating these exosomal markers with traditional serum markers and imaging techniques holds the promise of constructing a multi-modal early-warning and precision-monitoring system for CRLM.

Although liquid biopsy markers exhibit broad application prospects, their translation into routine clinical practice still faces numerous challenges. The principal bottlenecks hindering the clinical application of exosomes and ctDNA in CRLM early warning include inconsistent assay standardization, inter-platform heterogeneity of results, and the lack of unified cutoff values. Currently, ctDNA detection has not yet established unified standards for sample processing, sequencing platforms, and analytical pipelines, with marked heterogeneity of results across platforms; exosome isolation and purification methods vary substantially, and miRNA quantification is affected by the choice of internal references and normalization strategies, making diagnostic thresholds difficult to unify. Therefore, establishing standardized operating procedures and conducting multicenter, large-sample clinical validation to determine the optimal cutoff values for each marker constitute the key issues that urgently need to be addressed to advance their clinical application in CRLM early warning.

Traditional markers and imaging techniques together constitute the current cornerstone for the diagnosis and monitoring of colorectal cancer liver metastasis, as they can accurately assess the location, size, number, and morphological changes of metastatic lesions. However, traditional methods have certain limitations in detecting micrometastases, evaluating molecular heterogeneity, and achieving early warning. Liquid biopsy markers-including circulating tumor DNA (ctDNA), circulating tumor cells (CTCs), and exosome-carried nucleic acids and proteins-are progressively supplementing these deficiencies. Through non-invasive, dynamic monitoring of tumor-derived biological information in the blood, they hold the promise of achieving earlier metastatic warning and more precise therapeutic monitoring. The real-time tumor burden and dynamically evolving molecular characteristics reflected by these liquid biopsy markers also provide critical real-time biological evidence for the formulation of CRLM treatment strategies, particularly for the timing assessment of conversion therapy, the precise selection of targeted agents, and the identification of immunotherapy windows, thereby facilitating the transition of therapeutic decision-making from ‘static staging’ toward a ‘dynamic precision’ paradigm.

### 3.3 Metastatic biology and prognosis following hepatectomy

In CRLM, the clinical application of biomarkers should first distinguish between two related but distinct clinical questions: the first concerns the biology of liver metastasis formation and growth, with the core objectives being the prediction or early detection of hepatic metastasis, the assessment of metastatic burden and progression rate, and the prediction of systemic therapy (particularly immunotherapy) response; the second concerns prognosis following hepatectomy, with the core objective being the prediction of intrahepatic or extrahepatic recurrence and long-term survival in patients who have undergone curative-intent locoregional therapy. The corresponding clinical endpoints for these two questions are not identical: the former uses time to metastasis, time to progression, or systemic therapy response as primary endpoints, whereas the latter uses recurrence-free survival (RFS), overall survival (OS), and recurrence patterns as primary endpoints.

At the level of metastatic biology, the preferred assessment indicators include circulating tumor DNA (ctDNA), circulating tumor cells (CTCs) and their EMT/stemness characteristics, exosomal miRNAs, and indicators reflecting the pre-metastatic niche (PMN) or hepatic immune microenvironment. These indicators can dynamically reflect tumor shedding, clonal evolution, invasive and metastatic potential, and the status of the intrahepatic ‘soil,’ making them suitable for the early detection of minimal residual disease or subclinical hepatic metastasis and for monitoring systemic therapy response. At the level of post-hepatectomy prognosis, the preferred assessment indicators include resectability assessment, clinical risk scores (e.g., CRS score), CEA levels and their dynamic changes, RAS/BRAF status, future liver remnant (FLR), and R0 status, combined with pathological and clinical parameters such as tumor number and size and margin status, to comprehensively evaluate postoperative recurrence risk and long-term survival.

## 4 Therapeutic strategies for colorectal cancer liver metastasis

The management of CRLM has progressively evolved from a singular surgical resection model into a comprehensive, multidisciplinary system centered on multidisciplinary comprehensive treatment and stratified management according to tumor resectability and molecular characteristics. Overall, for patients with initially resectable disease, R0 resection should be pursued as the fundamental goal, with comprehensive optimization centered on resectability assessment, local techniques to enhance resectability, and perioperative systemic therapy; for patients with initially unresectable disease, it is necessary to distinguish between the ‘potentially convertible’ and ‘unresectable’ categories, with conversion therapy aiming to achieve surgical opportunity in the former, and systemic therapy combined with liver-directed locoregional therapy as the mainstay in the latter, with liver transplantation considered under rigorous selection (key clinical trials summarized in **Table 2**). Concurrently, emerging strategies such as cell therapy, cancer vaccines, and targeted metabolic–epigenetic and organ-specific immunomodulation are continuously expanding the therapeutic frontier, but their clinical value remains to be further validated.

**Table 2:** Key clinical trials evaluating systemic therapeutic strategies in colorectal cancer liver metastasis.

Trial	Population/molecular subgroup	Line of therapy	Comparator/design	Primary endpoint and effect size	Liver metastasis–specific findings
CRYSTAL (117)	KRAS wild-type mCRC	First-line	FOLFIRI ± cetuximab	ORR 57.3% vs 40.2%; PFS 9.9 vs 8.2 months	R0 resection rate ≈9.8% vs 4.5% in the liver-limited subgroup
CELIM (118)	Initially unresectable CRLM	First-line (conversion)	FOLFOX/FOLFIRI + cetuximab (single-arm)	ORR ≈68%; conversion-to-resection rate ≈34% (R0 ≈28%)	Directly reports conversion-to-resection endpoints in CRLM
BEACON (119)	BRAF V600E-mutant mCRC	≥ second-line	Encorafenib + binimetinib + cetuximab vs standard chemotherapy + cetuximab	OS 9.0 vs 5.4 months (HR 0.52); ORR 26% vs 2%	No separate CRLM subgroup reported
KEYNOTE-177 (120)	MSI-H/dMMR mCRC	First-line	Pembrolizumab vs chemotherapy	PFS 16.5 vs 8.2 months (HR 0.59); OS difference confounded by crossover	Liver metastasis analyzed as an exploratory subgroup, not a prespecified stratification
CheckMate 8HW (121)	MSI-H/dMMR mCRC	First-line/later-line	Nivolumab + ipilimumab vs chemotherapy; nivolumab + ipilimumab vs nivolumab	Combination vs chemotherapy: PFS HR 0.21; 24-month PFS 72% vs 14%	Combination showed greater benefit over monotherapy in the subgroup without liver metastasis
REGONIVO (122)	MSS/pMMR mCRC	≥ second-line	Regorafenib + nivolumab (single-arm phase I/II)	Overall ORR 36%	ORR 50% in lung metastasis vs 15.4% in liver metastasis
LEAP-017 (123)	Non–MSI-H mCRC	≥ second-line	Lenvatinib + pembrolizumab vs standard of care	OS HR 0.83 (not significant); PFS improved	Liver metastasis subgroup trend consistent with the overall population but without independent statistical power
ILOC/EORTC-1560 (124)	Unresectable liver-dominant mCRC	Later-line	Durvalumab + tremelimumab + local ablation/radiotherapy (single-arm phase II)	No objective response in non-treated lesions; median PFS 2.2 months	Direct negative result in CRLM
METASYNC (125)	Resectable synchronous CRLM	Perioperative	Simultaneous vs staged resection (RCT, n = 85)	2-year OS/DFS favored simultaneous resection; OS difference no longer significant at 47 months	Direct evidence on timing of surgery in CRLM
SECA-II (126)	Unresectable liver-limited CRLM (highly selected, n = 15)	Liver transplantation	Single-arm	1-/3-/5-year OS 100%/83%/83%; 3-year PFS 35%	Direct evidence for liver transplantation in CRLM
TransMet (127)	Unresectable liver-limited CRLM	Liver transplantation + chemotherapy vs chemotherapy alone (RCT)	Randomized controlled	ITT 5-year OS 56.6% vs 12.6%; PP 73.3% vs 9.3%	Direct randomized evidence for liver transplantation in CRLM
SIRT (128)	Chemotherapy-refractory/intolerant mCRC	Later-line	Y-90 resin microsphere SIRT vs best supportive care	OS HR 0.48 (95% CI 0.27–0.87)	Evidence for liver-directed therapy
DC vaccine phase II (129)	After complete resection of CRLM	Adjuvant	DC vaccine vs observation (randomized phase II)	Median DFS 25.3 vs 9.5 months	Direct evidence for adjuvant immunotherapy after CRLM resection

### 4.1 Treatment of initially resectable CRLM

For patients with CRLM who are eligible for surgery, R0 resection constitutes the core principle for achieving an optimal prognosis. Large-scale retrospective studies have shown that 5-year survival rates after complete resection can reach 30–57% (5), with actual 10-year survival rates of approximately 20% (130). However, surgical resection is not the sole pathway to long-term survival; selected patients may achieve significantly prolonged survival through ablation combined with resection or, under rigorous selection, liver transplantation.

#### 4.1.1 Key elements in the assessment of surgical resectability

In clinical practice, only 10–20% of patients fulfill the criteria for direct surgical resection at the time of diagnosis (6). The assessment of resectability requires comprehensive consideration of the following dimensions (131–134): Future liver remnant (FLR): an FLR of ≥20–25% is generally required for normal liver parenchyma, or ≥30–40% for compromised parenchyma (e.g., chemotherapy-associated steatohepatitis or fibrosis). FLR volume can be measured by CT or MRI, and functional assessment can be supplemented by hepatobiliary scintigraphy (e.g., ^99m^Tc-Mebrofenin) or ICG clearance testing. Vascular involvement: tumor invasion of or proximity to major hepatic veins, portal vein branches, or the inferior vena cava may limit resectability; however, some centers have attempted resection with reconstruction for involvement confined to the hepatic vein–inferior vena cava confluence. Biological resectability: even when technically resectable, if the tumor exhibits multifocal rapid progression, is refractory to systemic therapy, or is accompanied by uncontrolled extrahepatic metastases, the survival benefit of surgery is limited; molecular markers (RAS/BRAF mutation status, MSI status, etc.) aid in assessing biological aggressiveness. Technical resectability: comprehensive judgment based on multidisciplinary consultation, combined with intraoperative ultrasound, three-dimensional reconstruction, and other techniques to precisely evaluate the three-dimensional relationships between lesions and vascular/biliary structures.

#### 4.1.2 Local techniques to increase resectability

For patients initially deemed unresectable due to insufficient FLR, the following strategies may be employed to improve surgical opportunities (135–138): Portal vein embolization (PVE): embolization of the portal vein branches on the side to be resected induces compensatory hypertrophy of the contralateral liver lobe, typically increasing the FLR by approximately 10–15% within 4–6 weeks. Associating liver partition and portal vein ligation for staged hepatectomy (ALPPS): induces FLR hypertrophy more rapidly than PVE but is associated with significantly higher perioperative morbidity and mortality, necessitating strict control of indications. Two-stage hepatectomy: suitable for patients with bilobar multiple metastases; the first stage involves local resection or ablation of lesions (preserving the FLR side) combined with contralateral PVE, and once sufficient FLR hypertrophy is achieved, the second-stage radical resection is performed. Parenchymal-sparing hepatectomy: for patients with oligometastatic or recurrent metastases, wedge resection or subsegmentectomy is preferentially performed rather than extended hepatectomy to preserve more functional liver parenchyma and create conditions for potential repeat resection. Repeat hepatectomy: for intrahepatic recurrence of CRLM, if lesions are localized and FLR is sufficient, second or even third hepatectomy can still provide long-term survival benefit.

#### 4.1.3 Perioperative systemic therapy and surgical timing for resectable CRLM

For resectable CRLM, neoadjuvant chemotherapy (2–3 months preoperatively) provides a window for biological assessment of the tumor, but whether it can improve OS remains controversial; it is currently preferred for high-risk patients (e.g., CRS score ≥3, multiple metastases, RAS mutation).

Regarding surgical timing, the optimal surgical strategy for synchronous liver metastases (simultaneous versus staged resection) remains controversial. The METASYNC prospective controlled trial (125), comparing outcomes between the two groups, found that simultaneous resection yielded superior 2-year OS and DFS compared with staged resection, but the OS difference was no longer significant after 47 months. Given the trial’s small sample size and prolonged enrollment period, this conclusion requires validation in larger-scale, prospective, randomized controlled studies. In current clinical practice, the choice between simultaneous and staged resection requires comprehensive consideration: simultaneous resection avoids two operations and anesthesia risks but may increase postoperative complications; staged resection allows more adequate preoperative preparation and biological observation of the tumor.

### 4.2 Conversion therapy for potentially convertible (initially unresectable) CRLM

For initially unresectable CRLM, conversion therapy-whereby preoperative systemic therapy (chemotherapy combined with targeted agents) is administered to shrink tumors and achieve a transition from unresectability to resectability-constitutes a vital component of current clinical practice. High-quality conversion therapy can successfully convert approximately 15–30% of initially unresectable CRLM patients to a resectable state (118), with the CELIM study reporting a conversion resection rate of approximately 34% (R0 approximately 28%) (118). The core endpoints of conversion therapy are the conversion resection rate and R0 resection rate; after achieving conversion resection, RFS and OS are subsequently assessed according to the principles outlined in Section 4.1. Chemotherapy combined with anti-EGFR monoclonal antibodies in RAS wild-type patients and chemotherapy combined with bevacizumab in RAS-mutant patients constitute the principal conversion regimens.

### 4.3 Systemic therapy for unresectable CRLM (by molecular subgroup)

Systemic therapy is the central pillar of the comprehensive management framework for CRLM, encompassing chemotherapy, targeted therapy, immunotherapy, cell therapy, and cancer vaccines. In recent years, a series of high-quality clinical trials have continuously reshaped the treatment landscape for unresectable CRLM, delivering durable benefits to patients.

#### 4.3.1 Immunotherapy for MSI-H/dMMR mCRC

Immune checkpoint inhibitors (ICIs) restore anti-tumor immune responses by blocking inhibitory receptor signals on the T cell surface. MSI-H/dMMR tumors, owing to mismatch repair deficiency, harbor high tumor mutational burden (TMB) and high neoantigen burden with abundant T cell infiltration, providing an immunological basis for ICI response. The KEYNOTE-177 phase III trial established pembrolizumab as the first-line standard for MSI-H/dMMR mCRC: compared with standard chemotherapy, median PFS was significantly prolonged (16.5 months vs. 8.2 months), with fewer treatment-related adverse events (120). The CheckMate 8HW phase III trial further confirmed the efficacy of nivolumab combined with ipilimumab: dual-ICI combination significantly improved PFS compared with chemotherapy, and compared with nivolumab monotherapy, the combination was more beneficial in certain subgroups (121). It should be noted that the enrollment populations of these trials comprised mCRC overall, not CRLM specifically; the liver metastasis subgroup data were mostly exploratory analyses. MSI-H/dMMR is a molecular indication for immunotherapy rather than a liver metastasis-specific predictor of efficacy.

#### 4.3.2 Systemic therapy for MSS/pMMR mCRC

Systemic chemotherapy constitutes the cornerstone of MSS/pMMR mCRC treatment. Combination chemotherapy regimens based on fluoropyrimidines (5-FU/capecitabine) and combined with oxaliplatin (FOLFOX regimen) or irinotecan (FOLFIRI regimen) are the standard first-line options. In patients with RAS wild-type tumors, chemotherapy combined with an anti-EGFR monoclonal antibody (cetuximab or panitumumab) can further improve the objective response rate (ORR) and R0 resection rate (117); the CRYSTAL trial demonstrated that FOLFIRI combined with cetuximab can extend the median progression-free survival (PFS) of patients with KRAS wild-type mCRC from 8.2 months to 9.9 months (117). For patients with RAS-mutant tumors, chemotherapy combined with bevacizumab is the standard of care (139).

In terms of molecular subgroup-directed later-line/specific treatment: patients with BRAF V600E mutations have an extremely poor prognosis, and the BEACON trial established encorafenib combined with cetuximab as the standard later-line treatment for BRAF V600E-mutated mCRC (119). mCRC with HER2 overexpression/amplification can benefit from dual anti-HER2 therapy (trastuzumab combined with pertuzumab or lapatinib). In patients with KRAS G12C mutations, strategies combining sotorasib or adagrasib with anti-EGFR agents have shown promising activity in early-phase clinical trials.

#### 4.3.3 Immunotherapy and liver metastasis-specific immunomodulation in MSS/pMMR mCRC

More than 90% of mCRC belongs to the MSS/pMMR subtype, which shows almost no response to ICI monotherapy; the underlying mechanisms involve low TMB, low neoantigen load, and an ‘immune-cold’ TME (140). ICI combination strategies for MSS/pMMR currently constitute early clinical evidence and should not be conflated with the standard treatment for MSI-H/dMMR.

Regarding ICI combined with anti-angiogenic agents, the REGONIVO trial showed that regorafenib combined with nivolumab yielded an overall ORR of 36% in MSS/pMMR CRC, but only 15.4% in patients with liver metastases compared with 50% in those with pulmonary metastases (122); similar trends have been repeatedly observed in studies such as LEAP-017 (29, 123). These results provide indirect evidence that the hepatic microenvironment constitutes a barrier to immunotherapy resistance, but they all derive from subgroup/pooled analyses and are accompanied by confounding factors such as patient selection and molecular subtypes. The EORTC-1560-GITCG/ILOC phase II trial of ICI combined with local therapy observed no objective responses in non-target lesions, with a median PFS of only 2.2 months (124), suggesting that even when combined with local ablation/radiotherapy, the immunosuppressive microenvironment of liver metastases may be difficult to reverse readily.

### 4.4 Liver-directed locoregional therapy

For patients with unresectable, liver-limited CRLM, a variety of locoregional treatment modalities can serve as important complements or alternatives to systemic therapy, with stratified use according to therapeutic intent. Thermal ablation technologies, such as radiofrequency ablation (RFA) and microwave ablation (MWA), are suitable for patients with a limited number and small size of hepatic metastases (typically fewer than 3, with a diameter of less than 3 cm) and can achieve a high rate of local control comparable to surgical resection (141). Stereotactic body radiotherapy (SBRT) provides a non-invasive local control approach through precisely targeted high-dose radiation, making it particularly suitable for patients who are not candidates for surgery or ablation. Selective internal radiation therapy (SIRT) delivers high-dose β-radiation selectively to hepatic metastases by infusing microspheres loaded with the Yttrium-90 (Y-90) radioisotope into the hepatic artery; an updated network meta-analysis showed that for patients with chemotherapy-refractory or intolerant mCRC, Y-90 resin microsphere SIRT demonstrated a longer OS compared with best supportive care (HR 0.48; 95% CI 0.27–0.87) (128), and ESMO consensus guidelines currently position SIRT as a selective treatment option for patients in whom chemotherapy has been ineffective (142). The efficacy endpoints of liver-directed locoregional therapy should report liver-specific progression to distinguish its local control effect on intrahepatic lesions from its impact on the systemic disease process.

### 4.5 Application of liver transplantation in unresectable CRLM

For patients with liver-confined, unresectable CRLM, liver transplantation, as a potentially curative treatment modality, is undergoing a profound evolution in its clinical role and value, gradually transitioning from an exploratory, salvage attempt toward a therapeutic option that can yield long-term survival benefits under rigorous selection. In 2013, the SECA-I study from Oslo University Hospital in Norway (143) first reported: 21 patients with unresectable CRLM who underwent liver transplantation after strict selection (primary tumor resected with no lymph node or extrahepatic metastases, ECOG performance status 0–1, receipt of ≥6 weeks of chemotherapy) achieved 1-, 3-, and 5-year OS rates of 95%, 68%, and 60%, respectively, although the 1-year PFS was only 35%. The SECA-II study in 2020 adopted even more stringent inclusion criteria, with 15 patients achieving 1-, 3-, and 5-year OS rates of 100%, 83%, and 83% (median time to recurrence 13.7 months, 3-year PFS 35%), and proposed the Oslo scoring system (126) (0–4 points: tumor diameter >5.5 cm, CEA >80 μg/L, interval from primary resection to liver transplantation <2 years, and progression during chemotherapy, each item accounting for 1 point). The TransMet study randomized 94 patients into an intention-to-treat population, with 47 patients in the liver transplantation plus chemotherapy group and 47 in the chemotherapy-only group; the median follow-up was 59.3 months, and in the intention-to-treat population, the 5-year OS was 56.6% in the transplantation plus chemotherapy group and 12.6% in the chemotherapy-only group, while the per-protocol 5-year OS rates were 73.3% and 9.3%, respectively (127). This RCT provides robust evidence supporting the survival benefit of liver transplantation in rigorously selected patients with unresectable CRLM. In regions with donor scarcity, such as Asia, living-donor liver transplantation has yielded similar results (144); the RAPID technique (auxiliary partial orthotopic liver transplantation with partial segment grafts, followed by total hepatectomy after graft regeneration) has been confirmed as safe and feasible in multicenter studies (145).

Although the above studies have brought new therapeutic hope for highly selected patients with unresectable CRLM, the widespread application of liver transplantation still faces numerous fundamental limitations: the stringent inclusion criteria are difficult to fully replicate in clinical practice and currently apply only to highly selected patients without extrahepatic disease; the severe global shortage of donor organs directly limits accessibility; long-term survival data remain relatively scarce, and large-scale prospective long-term follow-up studies are insufficient, leaving its long-term efficacy, recurrence risk, and ultimate impact on OS uncertain, and thus it has not yet become a routine recommendation.

### 4.6 Cell therapy and cancer vaccines

Adoptive cell therapy (ACT) primarily encompasses chimeric antigen receptor T cell (CAR-T) therapy, TCR-engineered T cell (TCR-T) therapy, and tumor-infiltrating lymphocyte (TIL) therapy. A Phase I dose-escalation trial of CEA-targeted CAR-T cells demonstrated a favorable safety profile in mCRC patients, with approximately 60% of patients experiencing a significant decline in serum CEA levels after treatment (146); guanylyl cyclase C (GCC)-targeted GCC19CART has received FDA Fast Track Designation, with preliminary data showing a favorable safety profile and signals of antitumor activity (147). However, CAR-T therapy in solid tumors faces multiple obstacles, including antigen heterogeneity, tumor infiltration, and TME immunosuppression (148). In the field of TIL therapy, a patient with KRAS G12D-mutated mCRC achieved complete regression of metastatic lesions and a 9-month partial response following the infusion of CD8+ TILs targeting the KRAS G12D mutation (149), providing proof-of-concept for tumor-specific neoantigen-directed T cell therapy. Regarding dendritic cell (DC) vaccines, a randomized phase II clinical trial showed that patients receiving DC vaccine therapy following complete CRLM resection had an extended median DFS, from 9.5 months in the observation group to 25.3 months (129). The above strategies currently constitute early clinical evidence and should not be regarded as standard treatment until validated by confirmatory trials.

### 4.7 Emerging strategies targeting metabolism–epigenetics and organ-specific immunomodulation

With the progressive elucidation of the role of lactylation in CRC, strategies targeting lactate metabolism and lactylation are transitioning from proof-of-concept validation to clinical translation, but currently all remain preclinical concepts or very early clinical-stage investigations. LDHA inhibitors, such as GNE-140, can reduce lactate production and subsequently lower genome-wide lactylation levels; the MCT1 inhibitor AZD3965 is currently in Phase I/II clinical trials, aiming to block lactate transport and improve the immune microenvironment (150). However, non-selective metabolic inhibition may cause toxicity to normal tissues that depend on glycolysis, such as erythrocytes and skeletal muscle; thus, enhancing tumor selectivity and reducing systemic side effects are key challenges for the development of this class of drugs. Inhibitors of the ‘writers’ and ‘erasers’ of lactylation also show therapeutic potential: the p300/CBP inhibitor C646 has been shown in preclinical CRC models to reduce MRE11 lactylation levels, enhancing the efficacy of platinum-based drugs and PARP inhibitors (94); the KAT8 inhibitor MG149 suppresses CRC growth by inhibiting eEF1A2 lactylation-dependent protein translation (92). Regarding ‘erasers,’ because HDAC inhibitors simultaneously affect deacetylation and delactylation pathways, the development of highly selective lactylation modulators is a future priority.

In terms of immunotherapy sensitization, the combination of LDH inhibition with PD-1 blockade has shown synergistic antitumor activity in CRC mouse models (151), supporting the clinical development of this combinatorial strategy, but this constitutes preclinical evidence. Therapeutic strategies tailored to specific organ microenvironments-such as using macrophage/CAF/TGF-β/VEGF-targeted interventions for liver metastases and modulating neutrophils/CXCR2/NETs/STING for lung metastases-are emerging as new paradigms for overcoming organ-specific immunotherapy resistance, likewise constituting preclinical concepts. It should be particularly noted that the inherent metabolic differences among different metastatic organs are likely to profoundly influence the ultimate efficacy of such targeted metabolic interventions, and the clinical translational value of these strategies has not yet been established.

## 5 Organ-specific discrepancies: differential immunotherapy responses between hepatic and pulmonary metastases

### 5.1 Clinical evidence

In multiple retrospective cohort studies and exploratory subgroup analyses, the presence of liver metastases is associated with inferior clinical outcomes and reduced ICI efficacy (**Table 3**). Saberzadeh-Ardestani et al. (13) retrospectively analyzed dMMR mCRC patients receiving first-line pembrolizumab and found that, compared with patients without liver metastases (median PFS not reached), those with liver metastases had a significantly shorter median PFS (HR 2.6; P = 0.003). In MSS/pMMR mCRC, Chen et al. (14) reported a secondary analysis of a Phase II trial, showing that among MSS mCRC patients treated with durvalumab plus tremelimumab, the disease control rate (DCR) was 49% for those without liver metastases but only 14% for those with liver metastases (P = 0.03).

**Table 3:** Key mechanisms and therapeutic strategies in colorectal cancer liver metastasis.

Treatment strategy	Main experimental system	Human verification status	Clinical relevance	Limitations	Current Treatment Landscape
Pembrolizumab as first-line treatment for MSI-H/dMMR mCRC (standard treatment evidence)	Randomized controlled phase III trial (KEYNOTE-177) (113, 122)	Adequate; prospective RCT, primary endpoint PFS significantly improved (122)	Establishing the first-line standard treatment status for MSI-H/dMMR mCRC (122)	Long-term follow-up data are still being updated; interpretation of the crossover design with chemotherapy requires caution.	Has become the first-line standard for MSI-H/dMMR
Efficacy of pembrolizumab in the liver metastasis subgroup (metastasis-related evidence)	Retrospective cohort analysis; secondary analysis of randomized trials (13, 14)	Retrospective/exploratory subgroup evidence; shorter PFS in patients with liver metastases (HR≈2.6; P = 0.003) (13)	Suggests that liver metastasis may be associated with poorer ICI efficacy; this is correlative evidence, and confounding factors were not fully adjusted	Evidence comes from retrospective and exploratory subgroup analyses; no prospective stratification by metastatic site; confounding factors such as tumor burden and RAS/BRAF status exist.	Does not affect standard treatment selection, but suggests that patients with liver metastases may require more aggressive strategies
Nivolumab + Ipilimumab in MSI-H/dMMR mCRC (Standard of Care Evidence)	Randomized controlled phase III trial (CheckMate 8HW) (29)	Adequate; prospective RCT, PFS improved versus monotherapy (29)	Further improves efficacy compared with PD-1 monotherapy; establishes the status of dual immunotherapy combination (29)	Increased toxicity (irAE) of combination therapy; long-term OS data pending update.	Recommended by guidelines for MSI-H/dMMR
Efficacy of Nivolumab + Ipilimumab in the Liver Metastasis Subgroup (Metastasis-Related Evidence)	Exploratory subgroup analysis (29); retrospective pooled analysis (141)	Exploratory evidence; greater benefit in patients without liver metastases, limited benefit in those with liver metastases (29, 141)	Suggests that the liver microenvironment may weaken the efficacy of dual immunotherapy; still a hypothetical association	Small subgroup sample size; multiple baseline variables not adjusted; high heterogeneity across studies.	Not yet sufficient to change recommendations, but supports additional evaluation for patients with liver metastases
ICI Combined with Anti-Angiogenic Therapy in MSS/pMMR mCRC (Regorafenib/Lenvatinib + PD-1)	Early-phase clinical trials (REGONIVO (124), LEAP-017 (128))	Exploratory subgroup analysis; retrospective pooled analysis (141)	Higher response in lung metastases, extremely low response in liver metastases (ORR 0%–15%) (124, 141)	Mostly single-arm, small-sample studies; lack of prospective stratification design by metastatic site.	Not widely recommended, confirmatory studies are still needed
Local Therapies (SIRT/RFA/SBRT) Combined with Immunotherapy	Preclinical models; single-arm phase II trial (ILOC, etc.) (142)	Limited data from phase II trial; no abscopal effect observed (142)	Good local control, but systemic immune activation effect not established	Suboptimal optimization of combination regimens; difficulty in reversing the immune microenvironment.	Still in the experimental stage, not recommended for routine use
CAR-T Cell Therapy (Targeting CEA/GCC)	Humanized mouse models; phase I dose-escalation trial (143)	Phase I safety data; preliminary efficacy signal (126, 143)	Provides proof-of-concept for CAR-T in solid tumors	Antigen heterogeneity, difficulty in tumor infiltration, risk of off-target toxicity.	No approved products yet, early trials are ongoing at multiple centers
DC Vaccine as Adjuvant Therapy After Surgery	Mouse models; randomized phase II clinical trial (145)	Phase II trial showed prolonged DFS (9.5→25.3 months) (145)	Potential approach for preventing postoperative recurrence	Single-center, small sample size; multicenter phase III validation is required.	Not yet incorporated into routine adjuvant therapy

Fakih et al. (29) further consolidated this finding through a pooled analysis of multiple MSS mCRC trials: among patients receiving regorafenib plus nivolumab (± ipilimumab), the ORR was 56.3% in those with predominantly pulmonary metastases, but was 0% in patients with hepatic/peritoneal metastases. This discrepancy suggests that hepatic metastases may possess unique immunosuppressive characteristics that limit the efficacy of immunotherapy; however, since none of the relevant studies adequately balanced potential confounding factors, attributing this directly to the causal role of the liver microenvironment still requires caution.

Preclinical studies have suggested that the relationship between immunotherapy response and liver metastatic burden may be time-dependent. In a murine model, Yu et al. (28) found that the establishment of liver metastases is followed by systemic CD8+ T cell depletion. Accordingly, a hypothesis has been proposed that the ‘optimal window’ for PD-1 blockade may exist before the formation of liver metastases, and once metastasis is established and this depletion program is initiated, the efficacy of immunotherapy may be substantially attenuated. However, this hypothesis currently lacks direct validation in humans; moreover, clinically, not all patients with liver metastases exhibit primary resistance, and some patients with dMMR/MSI-H and concurrent liver metastases can still achieve durable responses.

### 5.2 Mechanistic differences

The physiological immunotolerant properties of the liver are considered a potentially important factor contributing to the resistance of hepatic metastases to ICIs and to the observed efficacy discrepancy. Due to continuous exposure to food antigens, microbial products, and low levels of endotoxins from the gut, the liver has evolved multi-layered mechanisms to suppress excessive immune activation (57). In liver metastases, this physiological immunoregulation is overexploited.

Structurally, the immune exclusion niche formed by the co-localization of SPP1+ TAMs and FAP+ CAFs, along with the TGF-β-driven, collagen-rich ECM barrier, physically ‘traps’ T cells at the tumor periphery (72). Functionally, macrophage-mediated systemic T cell elimination may play an important role: as previously discussed, FasL+ hepatic macrophages specifically induce the apoptosis of Fas+CD8+ T cells through Fas-FasL interactions, leading to the depletion of the systemic T cell pool and thereby depriving ICIs of their target (28).

In stark contrast, the pulmonary metastatic microenvironment generally exhibits a more pronounced inflammatory profile and a more favorable immune response milieu. Pulmonary metastases are enriched with T cells expressing higher levels of activation markers (such as CD27, CD44, ICOS, 4-1BB, and CD45RO), a higher density of antigen-presenting cells, and more extensive tertiary lymphoid structures (TLSs) (150). The pulmonary TME also demonstrates a higher level of cGAS-STING pathway activation, which promotes anti-tumor immunity by facilitating the production of Type I interferons, the activation of DCs, and the formation of TLSs (151). The presence of TLSs is correlated with an ‘immune-inflamed’ microenvironment, increased infiltration of CTLs and B cells, and improved immunotherapy responses (152).

Interestingly, immunosuppressive mechanisms also exist within pulmonary metastases-primarily driven by neutrophils with a pro-tumorigenic phenotype. These TANs suppress T cell responses through CD80/CD86-CTLA-4 signaling (153). However, a key advantage of pulmonary metastases is that the intrinsic function of T cells is not completely lost. They retain a certain degree of activation potential and effector function reserve, which can be re-engaged once the inhibitory signals are relieved by ICIs.

When both hepatic and pulmonary metastases coexist, the liver may play a more prominent role, negatively influencing immune responses at other sites through the aforementioned systemic T cell depletion mechanism. The study by Lee et al. (76) confirmed that the CTLA-4-high Treg-dependent immunosuppression induced by liver metastases can render anti-PD-1 monotherapy ineffective, even against extrahepatic metastases. Therefore, the treatment of patients with dual hepatic and pulmonary metastases requires a multidisciplinary strategy that differentiates the biological characteristics of metastases in different organs and targets the potential promoting role of liver metastases in systemic immune resistance with tailored interventions. Liver-directed local therapy (such as radiotherapy) combined with immunotherapy is thought to potentially reverse systemic immunosuppression by reducing intrahepatic immunosuppressive macrophages and restoring T cell survival (28).

When interpreting the above organ-specific efficacy discrepancies, it is also necessary to carefully consider multiple potential confounding factors. These include tumor burden, primary tumor location, RAS/BRAF and MSI/MMR status, prior treatment history, peritoneal or other site metastases, patient performance status (ECOG PS), lesion measurability, and the inclusion in some studies of patient populations with predominantly pulmonary lesions and relatively indolent biological behavior. These factors may independently affect ICI efficacy, independent of the metastatic organ itself: for example, patients with MSI-H or high TMB respond better to ICIs, right-sided colon cancer has a poorer prognosis and differential treatment response, and the inclusion of relatively indolent patient populations with predominantly pulmonary metastases may introduce selection bias. Without adjustment, these confounding factors may affect the independent role of the organ microenvironment in immune resistance.

In summary, the immunosuppressive characteristics of the liver microenvironment may contribute to the development of resistance, but translating these mechanisms into clinical predictive markers or therapeutic targets still requires further investigation. In particular, the frequency, timing, and quantitative impact of systemic T cell depletion on immunotherapy efficacy in human CRLM patients currently lack high-quality prospective research evidence; existing clinical evidence largely derives from retrospective subgroup analyses and single-arm trials, and therefore related conclusions still require further experimental validation.

## 6 Challenges and future directions

### 6.1 Tissue acquisition and spatial heterogeneity

In the mechanistic research and clinical decision-making for CRLM, tissue acquisition methods and the spatial heterogeneity they mediate constitute core scientific issues that cannot be overlooked. Significant molecular heterogeneity exists between primary tumors and liver metastases, between different regions within the same lesion, and among different metastatic lesions, and this heterogeneity directly affects the interpretation of biomarker detection results and the formulation of clinical treatment strategies.

Primary tumors cannot fully represent the molecular characteristics of liver metastases. CRC undergoes clonal selection and continuous evolution during metastatic progression; the subclones driving metastasis in the primary tumor may only achieve clonal dominance in the metastatic site, and new mutations not detected in the primary tumor may even emerge. Therefore, relying solely on molecular testing of the primary tumor to predict the biological behavior of liver metastases or to guide targeted therapy carries an inherent risk of inadequate representativeness.

Furthermore, needle biopsy and surgical resection specimens differ significantly in the depth of detection information and the timing of sample acquisition. Needle biopsy has the advantages of minimal invasiveness and repeatable sampling but is limited by the small sample volume, making it difficult to comprehensively resolve intralesional heterogeneity, and is susceptible to sampling bias that may miss key molecular events; surgical resection specimens can provide more complete histological and molecular information but can only be obtained after tumor resection, and therefore cannot provide real-time evidence for pre-treatment clinical decision-making. In addition, needle biopsy itself carries potential risks such as hemorrhage and needle-tract seeding, which also limit the clinical application of repeat biopsy.

Clear inter-lesional heterogeneity exists among multiple hepatic metastases. Multiple hepatic metastases in the same patient may carry different mutation profiles and transcriptomic characteristics, and their responses to the same therapeutic regimen may also differ. Treatment strategies formulated based on molecular testing of a single lesion may miss resistant clones already present in other lesions, thereby leading to therapeutic resistance and failure. Dynamic sampling before and after treatment is crucial for elucidating the molecular mechanisms of acquired resistance in CRLM. Pre-treatment baseline specimens mainly reflect the molecular characteristics of initially treatment-sensitive clones, whereas post-treatment specimens can reveal resistant clones enriched under therapeutic selection pressure and their driving mechanisms. However, repeat biopsy after treatment faces multiple challenges in clinical practice, including feasibility, patient compliance, and safety, and the acquisition of paired pre- and post-treatment samples is particularly difficult.

In summary, future research needs to adopt multi-lesion, multi-timepoint, multi-region sampling strategies, combined with liquid biopsy and other technical approaches, to systematically resolve the spatial and temporal heterogeneity of CRLM, thereby enhancing the reliability of molecular marker detection and the precision of clinical treatment decision-making.

### 6.2 Treatment-induced evolution and microenvironmental remodeling

Existing descriptions of the CRLM microenvironment predominantly reflect the pre-treatment tumor microenvironment, whereas treatment itself can significantly remodel the tumor and its immune microenvironment, rendering the ‘baseline microenvironment’ distinct from the ‘on-treatment/post-treatment microenvironment.’ Chemotherapy can induce DNA damage responses and immunogenic cell death, altering T cell infiltration, but may also select for resistant clones and upregulate immune checkpoint molecules (94, 95, 99); anti-VEGF agents improve drug delivery through vascular normalization and reduction of interstitial fluid pressure, and can reduce the infiltration of immunosuppressive myeloid cells and reshape T cell distribution (40, 139); anti-EGFR therapy can alter RAS downstream signaling dependency and clonal composition, and affect tumor-stromal interactions (114, 117); liver-directed interventions (resection, ablation, SIRT, SBRT) release tumor antigens and damage-associated molecular patterns through local tissue injury, potentially activating innate immune pathways such as cGAS-STING (71, 153), but may also induce compensatory immunosuppression (e.g., local hypoxia, macrophage and Treg accumulation) (28, 76, 124).

### 6.3 Limitations of current research

Despite significant advances in the mechanistic understanding and clinical management of CRLM, several important limitations must be acknowledged and addressed.

Limitations in the level of clinical evidence. The existing clinical evidence on organ-specific differences in ICI efficacy is derived mainly from exploratory subgroup analyses, retrospective cohorts, or early single-arm studies, and is predominantly small-scale and single-center, rather than from prospective randomized controlled trials stratified by metastatic site. These studies have limited sample sizes and statistical power and may be confounded by selection bias, heterogeneity of treatment regimens, and differences in prior therapies. Thus, although the existing evidence consistently points to an association between liver metastases and primary ICI resistance and poor prognosis, future clinical trials must be designed to prospectively stratify patients by metastatic site and report organ-level objective response rates to confirm these findings and guide organ-specific therapeutic decision-making.

Limitations in tumor heterogeneity. The biological heterogeneity of mCRC complicates the interpretation of organ-specific microenvironmental influences. Intrinsic molecular characteristics of tumor cells (e.g., TMB, neoantigen load, activity of the IFN-γ signaling pathway, antigen presentation capacity) may influence ICI responses independently of the metastatic organ. Moreover, clonal evolution during tumor progression can lead to molecular disparities between different metastatic sites; equally important, primary colorectal tumors and matched liver metastases may exhibit significant differences in genomic, transcriptomic, epigenetic, and immune microenvironmental profiles, and extrapolating the biological behavior of liver metastases based on primary tumor molecular features carries uncertainty, further adding to the analytical complexity.

Limitations in experimental models. Much of the mechanistic understanding of CRLM is derived from cell lines and animal models. While these studies have provided critical insights into the cellular and molecular aspects of metastatic colonization, translating these findings to human disease still faces challenges related to species-specific differences, the complexity of human immune responses, and tumor heterogeneity. Furthermore, existing models (e.g., intrasplenic or portal vein injection models) often fail to fully recapitulate the complete cascade of CRLM events-from PMN formation to CTC intrahepatic arrest, extravasation, and colonization-which largely limits the identification and validation of early-stage anti-metastatic targets.

Limitations in molecular mechanism validation and technology. Although research on lactylation in CRC is advancing rapidly, the field faces several key bottlenecks. The specificity and sensitivity of detection technologies for lactylation modifications, particularly in distinguishing L-lactylation from D-lactylation at the whole-proteome level, require improvement. Additionally, the crosstalk network between lactylation and other post-translational modifications such as acetylation, methylation, and phosphorylation remains almost entirely unexplored: given that lactylation and acetylation share many ‘writer’ and ‘eraser’ enzymes, understanding how their competitive and synergistic relationships influence CRC progression and therapeutic response is a critical question awaiting resolution. Concurrently, several recently reported molecular axes (such as those involving lactylation, the microbiota, and the PMN) still lack sufficient independent replication, and their robustness and generalizability need to be further confirmed in larger sample sizes and independent cohorts.

Limitations in review methodology. This review is narrative in nature, and the study selection process lacks systematicity; it did not adopt the pre-registration protocols, dual independent screening, and standardized risk-of-bias assessment procedures required by systematic reviews, so the inclusion of literature may be subject to selection bias. Meanwhile, influenced by publication bias, published studies tend to report positive results, and the emphasis of this review on positive studies may overestimate the effects of certain intervention strategies or molecular mechanisms, while negative or null results may be underestimated.

### 6.4 Future research directions

Looking ahead, the following directions merit prioritized attention and in-depth investigation:

Integration of multi-omics technologies to dissect the spatiotemporal heterogeneity of CRLM. The combined application of high-resolution technologies, including single-cell RNA sequencing (scRNA-seq), spatial transcriptomics, single-cell ATAC-seq, and multiplexed imaging, promises to systematically unveil the cellular composition, intercellular communication networks, and the spatial distribution of functional states in CRLM at single-cell resolution. Such information is crucial for identifying key cell subsets and signaling pathways that drive immunosuppression and for developing more precise targeting strategies.

Development of organ-specific therapeutic strategies. Given the significant disparities between liver and lung metastases in terms of the immune microenvironment and ICI response, future clinical trials should explore adaptive therapeutic strategies based on the site of metastasis. For patients with liver-predominant disease, priority should be given to combinatorial strategies that target the macrophage/CAF/TGF-β pathway, or liver-directed local therapy combined with immunotherapy. For those with lung-predominant metastases, exploration of ICIs combined with neutrophil/CXCR2 inhibition or STING agonists may be more appropriate.

Advancing the translation from omics discovery to clinically interpretable modeling and prospective validation. Multi-omics complexity per se does not equate to clinical utility; integrating molecular, imaging, and clinical data into interpretable, actionable decision tools, and measuring their decision-increment value through patient-centered outcomes, is the key to realizing precision therapy (154). Methodological studies conducted in gastric cancer have articulated the relevant principles: multimodal modeling for clinically meaningful risk integration, interpretable multimodal fusion for recurrence prediction, robust patient-level modeling with independent feature-marker validation, longitudinal molecular monitoring cited as a methodological template, and prospective multimodal validation for metastatic risk (155–158). Any CRLM subtype or feature marker must undergo external validation in independent prospective cohorts before clinical use. Precision therapy claims should be based on a clear and verifiable algorithm comprising a biomarker (e.g., RAS/BRAF/MSI status and ctDNA dynamics), a defined threshold, a therapeutic intervention, a timing selection, and patient-centered outcome measures (e.g., conversion to resectable status, recurrence-free survival, quality of life). For example, for patients with initially unresectable liver-confined CRLM, chemotherapy-based conversion could be guided by RAS/BRAF status and ctDNA clearance, with resectability reassessed at specific timepoints while monitoring quality of life and survival.

Targeting the PMN to prevent metastasis. Preventing the onset of metastasis is more effective and controllable than treating established metastases. Strategies such as exosomal integrin antagonism (e.g., cilengitide), NETs inhibitors (e.g., PAD4 inhibitors or DNase I), and agents that remodel the hepatic immune microenvironment (e.g., STING agonists) have all shown metastasis-preventive potential in preclinical models and merit further clinical development. However, translating these interventions from models to the adjuvant setting for high-risk CRC patients requires addressing critical issues of safety, timing of administration, and patient stratification using biomarkers.

Deciphering the lactylation regulatory network to overcome therapeutic resistance. Future efforts must develop highly selective, site-specific intervention strategies targeting lactylation-such as PROTAC technology to degrade specific lactyltransferases, or nano-targeted drug delivery systems directed at specific substrate protein lactylation sites-in order to precisely eliminate tumor-promoting lactylation events without disrupting normal metabolic and epigenetic regulation. Concurrently, elucidating the distinct functional roles and regulatory mechanisms of D-lactylation versus L-lactylation in CRC is another frontier that urgently needs to be conquered.

Strategies that modulate the gut microbiota, such as fecal microbiota transplantation (FMT), probiotic/prebiotic interventions, and targeted antibacterial therapy, have shown potential to synergize with ICI therapy in translational research, possibly by reshaping the tumor immune microenvironment and enhancing anti-tumor immune responses. At the individualized treatment level, the composition and functional characteristics of the gut microbiota (e.g., the relative abundance of specific genera, microbial metabolites, and related functional pathways) have the potential to serve as predictive biomarkers for ICI response, resistance, and immune-related adverse events, thereby assisting in the stratification and therapeutic decision-making of CRC/CRLM patients. Some studies have suggested correlations between specific microbiota signatures and ICI efficacy, but existing evidence largely derives from correlation analyses or small-sample exploratory studies, which cannot establish causal relationships between specific microbiota alterations and ICI efficacy. Meanwhile, gut microbiota composition is influenced by multiple factors, including geographic environment, diet, drug exposure, sample type, sequencing platform, and analytical pipeline, and existing studies exhibit significant heterogeneity and variability, limiting the generalizability and reproducibility of microbial markers. Therefore, before microbial markers have been prospectively validated, they should not be used alone as a basis for patient selection or treatment adjustment. Future research should combine multi-omics analyses and prospective clinical trials to clarify the actual predictive value, applicable populations, and standardized detection methods of microbial markers in patient stratification, providing a reliable basis for microbiota-based individualized treatment. At the combination therapy level, strategies that modulate the gut microbiota-including FMT, probiotic/prebiotic interventions, and targeted antibacterial therapy-have shown potential to synergize with ICI therapy in translational studies, possibly through mechanisms such as modulating drug metabolism, affecting dendritic cell and T cell function, reshaping the tumor immune microenvironment, and enhancing anti-tumor immune responses, and may offer new approaches for reversing immunotherapy resistance. However, the application of these interventions in CRC, particularly CRLM, remains investigational and should not be regarded as clinical protocols that can be directly adopted. In terms of safety, FMT carries potential risks of transmitting pathogens or multidrug-resistant bacteria, and the long-term ecological impact of microbial community interventions on the host microecosystem remains unclear; in the absence of prospectively validated microbial targets, antibacterial therapy may not only fail to provide benefit but may also exacerbate dysbiosis or cause other harm. Therefore, the combination of microbiota modulation with ICIs should be premised on validated microbial targets, standardized treatment protocols, and adequate safety assurance. Future research needs to further dissect the causal relationships between specific bacterial strains and specific proteins, elucidate their regulatory targets, signaling pathways, and specific effects on tumor cell metabolism and epigenetic status, and systematically evaluate the optimal timing, dose, duration, and benefit-risk ratio of microbiota interventions combined with ICIs, thereby developing more efficient and personalized combination therapeutic strategies.

## 7 Conclusions

Colorectal cancer liver metastasis is a complex biological process involving multi-dimensional, multi-step, and multi-signal crosstalk regulation. From the pre-conditioning of the hepatic microenvironment by primary tumor-derived extracellular vesicles to form the PMN, to the acquisition of invasive and disseminative capacities by CTCs through EMT and their arrival in the liver via the ‘mesenteric-portal circulation,’ to the dynamic interactions among tumor, stromal, and immune cells within the hepatic TME, and the systemic regulation by the gut microbiota and metabolic reprogramming-particularly lactylation-as ‘environmental modifiers’-this cascade of events collectively constitutes the landscape of CRLM initiation and progression. At the clinical level, multidisciplinary comprehensive management centered on surgical resection and combined with chemotherapy, targeted therapy, immunotherapy, and locoregional therapy is the fundamental framework for the current management of CRLM. However, the inherent immunotolerant characteristics of the liver may represent an important cause of primary resistance of hepatic metastases to immunotherapy, a finding that challenges the indiscriminate application of immunotherapy strategies and underscores the necessity of a precision medicine mindset grounded in the organ microenvironment.

Looking forward, the research and management of CRLM are at an early exploratory stage of translating molecular mechanistic insights into clinical precision interventions. The deep application of single-cell and spatial multi-omics technologies, and the functional dissection of emerging regulatory layers such as lactylation and the microbiota, hold promise for further elucidating the molecular basis of CRLM and revealing new potential intervention targets. However, most current studies remain at the preclinical or early exploratory stage, and their efficacy, safety, and clinical translational value in humans have not been confirmed. The translation from mechanistic discovery to clinical application still requires overcoming multiple obstacles, including species differences, individual heterogeneity, intervention timing and dose selection, and long-term safety assessment. Therefore, rigorously designed prospective clinical studies are urgently needed to systematically validate the above strategies, thereby facilitating the transition of CRLM management from ‘empirical therapy guided by metastatic site’ toward ‘mechanism-driven precision therapy.’
